# Evolution Under Competition Increases Population Production by Reducing the Density‐Dependence of Net Energy Fluxes and Growth

**DOI:** 10.1002/ece3.71071

**Published:** 2025-03-17

**Authors:** Charlotte L. Briddon, Ricardo Estevens, Giulia Ghedini

**Affiliations:** ^1^ GIMM—Gulbenkian Institute for Molecular Medicine (Previously Instituto Gulbenkian de Ciência) Lisbon Portugal; ^2^ School of Biological Sciences Monash University Clayton Australia

**Keywords:** communities, metabolism, photosynthesis, respiration, species interactions

## Abstract

Competition can drive rapid evolution, but forecasting how species evolve in communities remains difficult. Life history theory predicts that evolution in crowded environments should maximize population production, with intra‐ and inter‐specific competition producing similar outcomes if species compete for similar resources. Despite its appeal, this prediction has rarely been tested in communities. To test its generality and identify its physiological basis, we used experimental evolution to maintain four species of marine phytoplankton alone or together in a community for 4.5 months. We then quantified changes in their metabolism, demography, and competitive ability at two timepoints (~60 and 120 generations) in common garden experiments. One species was outcompeted during the evolution experiment. For the other three, we found the same evolutionary outcome: species evolved greater biovolume production regardless of competition treatment but did so either by increasing max. population size or individual cell size. Biovolume production increased because of the differential evolution of photosynthesis and respiration under intense competition. These metabolic changes meant that intraspecific competition decreased, and cells maintained higher rates of net energy production and growth as populations neared the stationary phase. Overall, these results show that intra‐ and inter‐specific competition influence physiological and population parameters similarly in species that compete for essential resources. Life history theory thus provides a valuable base for predicting how species evolve in communities, and our results show how these predictions relate to the evolution of metabolism and competitive ability.

## Graphical abstract

Using experimental evolution, we determined the impact of intra‐ versus interspecific competition on three marine phytoplankton species. We found that the species achieved the same evolutionary outcomes, increasing their biovolume production due to the differential evolution of photosynthesis and respiration. Overall, we show that competition for essential resources leads to highly predictable changes in physiology and competitive ability that evolve in parallel to drive demography.

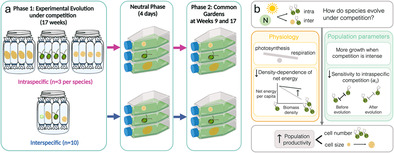

## 1 Introduction

Biodiversity change can drive rapid evolution because species introductions or losses alter competition regimes (Santamaría and Méndez 2012). However, which species traits evolve and how they evolve in response to competitive interactions remains difficult to anticipate (Aubree et al. 2020; Castledine et al. 2020).

Macroevolutionary patterns indicate that, when species compete for similar resources, they can evolve to either become more different (character displacement) or more similar (character convergence) (Fox and Vasseur 2008; Pfennig and Pfennig 2009). In the case of character displacement, the evolved trait differences should promote coexistence by increasing niche differences (Stuart and Losos 2013). But theory suggests that opportunities for niche differentiation are limited when species compete for essential resources; thus, competitive ability may be more likely to evolve in such cases (Fox and Vasseur 2008; Hart et al. 2019). Recent empirical work on plants and algae confirms these theoretical predictions: evolution in response to interspecific competition can result in a more efficient use of resources (e.g., lower minimum resource requirement [Bernhardt et al. 2020; Germain et al. 2020]), thus increasing competitive ability (Hart et al. 2019). However, requirements for different resources are linked through complex metabolic pathways (are not independent) (Sunday et al. 2024; Thomas et al. 2017) and any trait change can affect niche and fitness differences simultaneously (Gallego et al. 2019; Song et al. 2019). So, assessing which resource use traits evolve (e.g., minimum nitrogen requirement) can indicate the driver of selection but does not necessarily facilitate predictions of evolution (Gallego and Narwani 2022).

An alternative approach could be to study density‐dependent processes of population regulation, which are nearly ubiquitous in nature (Mallet 2012). Thinking about evolution under competition in terms of changes in density‐dependence can help generalise predictions across species and connect ecology with life history theory (Sakarchi and Germain 2023). An advantage of life history theory is to provide a clear prediction about the quantity that should be maximised under competition. Originally, MacArthur predicted that evolution in a stable and crowded (resource‐limited) environment should maximise the equilibrium size of a population (carrying capacity; *K*) (MacArthur 1962; Macarthur and Wilson 1967). Subsequent refinements of these predictions show that, when the environment fluctuates (i.e., there is variation in the strength of density‐dependence), both population intrinsic growth rate (*r*) and *K* are maximised—thus competition should select for greater population production (Lande et al. 2009; White and Marshall 2023). Despite its appeal, there are few empirical tests of this prediction in response to intraspecific competition (Bierbaum et al. 1989; Engen and Sæther 2016), and almost none under interspecific competition (Ghedini and Marshall 2023).

The lack of tests may partly be explained by the controversy regarding ideas of *r*–*K* selection (Sakarchi and Germain 2024). The assessment of the parameter *K* in particular poses some challenges because, in the *r*–*K* formulation of the logistic growth model, *K* is treated as an independent biological parameter, but in fact, *K* is not independent from *r* (Sakarchi and Germain 2024; Marshall et al. 2023):

(1) $$\frac{\mathrm{dN}}{\mathrm{dt}}=r\left(1-\frac{N}{K}\right)N$$

This issue can be resolved by using growth models with explicit intraspecific competition coefficient (α), such as the original formulation of Verhulst (Verhulst and Delphenich 1838), because they emphasize the underlying demographic processes of growth and energy use (even though both models describe the same dynamics) (Mallet 2012; Marshall et al. 2023; Fronhofer et al. 2024):

(2) $$\frac{\mathrm{dN}}{\mathrm{dt}}=\left(r-\mathrm{\alpha N}\right)N$$

We test the predictions of life‐history theory under both intra‐ and interspecific competition using marine phytoplankton as a model system. We further attempt to explain these evolutionary responses by connecting population parameters with metabolic traits (i.e., photosynthesis, respiration and net energy production as their difference over 24 h). Demographic responses should covary with metabolism because energy fluxes influence growth and competitive ability (Bassar et al. 2013; Pettersen et al. 2020; Auer et al. 2018). Since metabolic rates can evolve rapidly (Padfield et al. 2016; Alton and Kellermann 2023), their assessment should help identify the physiological basis of evolved changes in competitive ability and demography.

**FIGURE 1:**
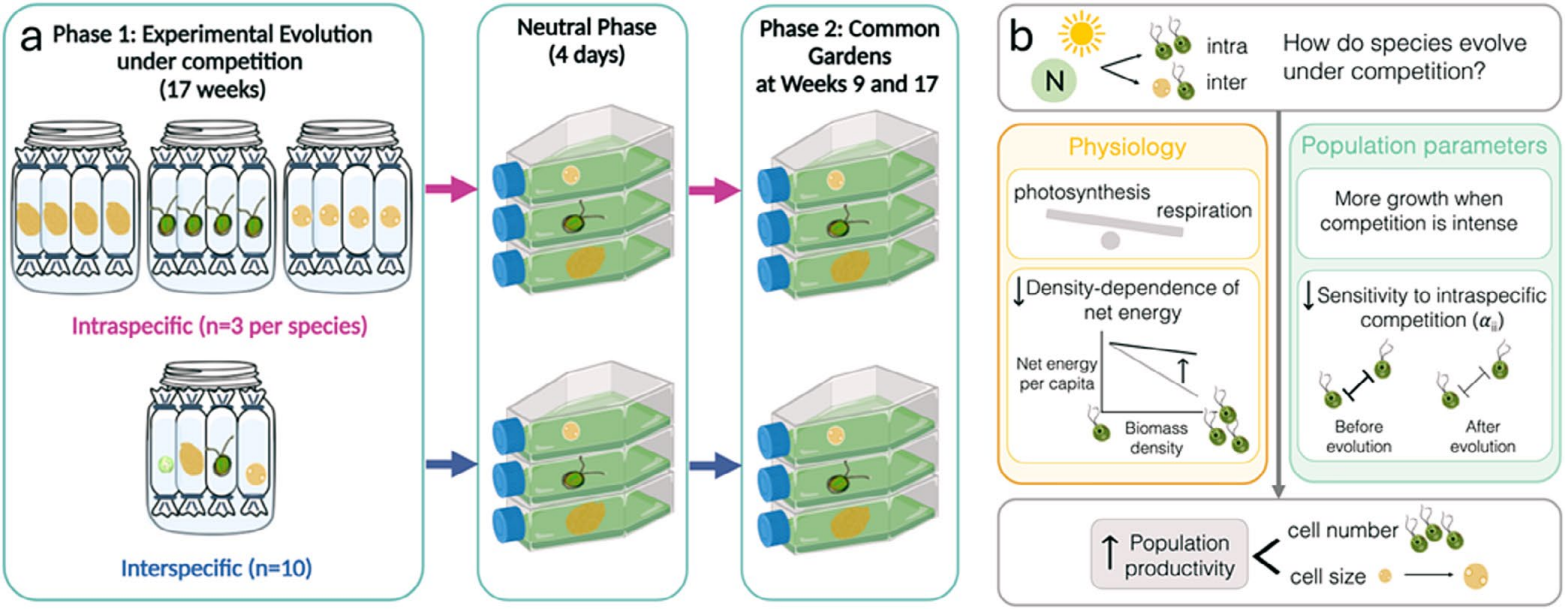
(a) Schematic showing the experimental set‐up of Phase 1 (Experimental Evolution) and Phase 2 (Common Gardens). During Phase 1, we evolved three species (*Amphidinium*, *Dunaliella*, *Tisochrysis*) either under intraspecific (monoculture) or interspecific competition (polyculture) for 4.5 months. At two time points (~60 and 120 generations), we used common garden experiments (Phase 2) to assess the evolution of each species. Before each common garden, we had a phase of neutral selection (4 days) to remove any plastic response. Created in BioRender. https://BioRender.com/y51g256 (b) Conceptual figure showing how evolution in response to competition influences phytoplankton physiology and population parameters.

We study phytoplankton because these species have rapid generation times (~1 doubling per day [Arin 2002]) and high population densities with substantial capacity for rapid evolution (Padfield et al. 2016), with adaptation generally occurring within a few weeks (Bach et al. 2018; Collins et al. 2014). We used four species that belong to different algal groups, thus encompassing distinctive traits in terms of morphology, size, and resource acquisition (Gallego et al. 2019; Hillebrand et al. 2022). We then grew these species either alone (intraspecific) or together in a community (interspecific competition). Each species was ‘caged’ in a dialysis bag to allow nutrient competition but keep species separate (Ghedini and Marshall 2023; Pomati et al. 2017; Poulson‐Ellestad et al. 2014; Reed and Martiny 2013; Scheuerl et al. 2020). One species was outcompeted and thus not included in the analyses (details in Section 2). For the remaining three species, we quantified changes in metabolism, demography, and morphology at two time points in common garden experiments (after 9 and 17 weeks of evolution, corresponding approximately to 60 and 120 generations [Arin 2002]). Our goals are (Figure 1b) to (1) determine if species evolve in the same way in the presence of intra‐ and interspecific competitors, that is, maximize population production as predicted by life history theory; (2) identify the physiological changes that underpin population responses, including the evolution of population parameters (growth rate, sensitivity to intraspecific competition); and (3) test if different species show the same evolutionary trajectories and underlying mechanisms of evolution.

## 2 Materials and Methods

To test the effects of intra‐ and interspecific competition on the evolution of metabolism, morphology and demography, we used four species of marine phytoplankton ( *Amphidinium carterae* RCC88 [dinoflagellate], *Dunaliella tertiolecta* RCC6 [chlorophyte], *Tisochrysis lutea* RCC90 [haptophyte] and *Nannochloropsis granulata* RCC438 [ochrophyte]) acquired from the Roscoff Culture Collection, France. These species belong to different taxonomic groups with varying cell sizes (*Amphidinium* = 949 ± 15 μm^3^; *Dunaliella* = 624 ± 4 μm^3^; *Tisochrysis* = 86 ± 0.66 μm^3^; *Nannochloropsis* = 19 ± 0.17 μm^3^). These species had been growing individually for years in the culture collection and in our lab, so they have not experienced interspecific competition for thousands of generations.

To determine how these species evolve in the presence of intra‐ or inter‐specific competitors, we grew these species either alone (intraspecific competition; ‘monoculture’) or together (interspecific competition; ‘polyculture’) for 4.5 months (Phase 1: Evolution experiment ~120 generations; Figure 1a). We then quantified trait evolution for each species at two time points using common garden experiments (Phase 2, done after 9 and 17 weeks of evolution corresponding to generation ~60 and ~120 respectively; Figure 1a). Common garden experiments involve placing populations exposed to different selective pressures (in our case intra‐ vs. inter‐specific competition) in the same, ‘common’ environment (in our case, a cell culture flask without interspecific competitors) to quantify evolved trait changes rather than plastic responses to the environment of evolution (De Villemereuil et al. 2016).

### 2.1 Phase 1: Experimental Evolution

We used dialysis bags cut to approximately 25 cm in length (MWCO 14 kDa, pore size 25 Angstrom, Dialysis membrane Membra‐CEL, Carl Roth, Germany) to ‘cage’ each population and keep species together in the same beaker but physically separate (Figure 1a). Bags were closed with a knot at the bottom and a nylon clip at the top, thus reducing their length to ~15 cm before being placed in 500 mL beakers. The dialysis bags allow competition for light (primarily within bags) and nutrients (within and between bags) but maintain physical separation between species (Ghedini and Marshall 2023; Pomati et al. 2017; Poulson‐Ellestad et al. 2014; Reed and Martiny 2013; Scheuerl et al. 2020), we could phenotype each independently in the common garden experiments (similar to Ghedini and Marshall 2023). Before starting the experiment, we grew a large volume (~2 L) of each species (strain) in isolation and used this same well‐mixed culture (the ancestor) to fill each bag of that species.

For the intraspecific treatment (monoculture), we set up three replicate beakers per species. Each beaker contained four dialysis bags, each filled with the same conspecific strain surrounded by enriched seawater medium (f/2 media prepared from 0.2 μm filtered and autoclaved natural seawater [Guillard and Ryther 1962]) containing no phytoplankton. This resulted in 12 dialysis bags split over three beakers for each species (Figure 1a). For the interspecific treatment (polyculture), we set up 10 replicate beakers using the same design, but each of the four dialysis bags was filled with a different species (Figure 1a). Independently of the treatment, each dialysis bag had the same initial biovolume of 5.2 × 10^9^ μm^3^, filled to a volume of 45 mL with f/2 media (biovolume density = 1.16 × 10^5^ μm^3^/μL; for reference the carrying capacity is ~10^6^ μm^3^/μL). Each beaker was filled to 500 mL with fresh media to submerge the bags.

We propagated the populations with a serial batch transfer (McDonald 2019). Once a week, we transferred a set volume of 25 mL from each dialysis bag to a new sterilised bag and beaker. The contents of the bag were topped up with 20 mL of fresh media to maintain the constant total volume of 45 mL. We also replaced the media in the beaker. Thus, each week the populations experienced fluctuating conditions, from lower competition (lower densities, high nutrients) just after the transfer to progressively higher competition towards the end of the week (higher densities, low nutrients). We chose this transfer frequency to strike a balance between imposing competition and allowing reproduction (and thus evolution) (Ghedini and Marshall 2023).

To facilitate the exchange of nutrients between bags, all beakers were bubbled daily with filtered atmospheric air (0.22 μm; Minisart, Sartorius, Göttingen, Germany) for 15 min every hour. For weeks 5–10, we reinoculated all bags with a lower set volume of 15 mL (instead of 25 mL) due to the high cell density to reduce nutrient limitation. After week 7, the populations of *Nannochloropsis* began to decline, particularly in the interspecific (poly) treatment, so this species was not included in the second common garden (week 17) and therefore was removed from all analyses. Light intensity was set at 60 μmol m^−2^s^−1^ with a 12:12 light: dark schedule using low‐heat 50 W led flood lights (Surface Luminária LED 230 V, Robert Mauser, Portugal). The temperature was maintained at 20°C ± 1°C.

### 2.2 Phase 2: Common Garden (CG) Experiments

After 9 and 17 weeks of evolution, we characterized the evolved lineages to determine changes in their traits. Specifically, we measured morphological, metabolic, and demographic traits to determine how these traits evolved in response to intraspecific (monoculture) and interspecific competition (polyculture). We tested two bags from each beaker for the mono treatment (*n* = 3 per species as the two bags were not independent) and all bags for the poly treatment (*n* = 10 for each species). Before beginning each CG experiment, we placed a 10 mL sample of each bag in a neutral environment (i.e., cell culture flask filled up to 100 mL with f/2 media) for 4 days (~4 generations) to remove any environmental conditioning. Removing these 10 mL from the dialysis bags did not affect the selective pressure of the experimental evolution (which was ongoing) because these samples were taken on the day of the batch transfer; thus, they were leftovers of the transfer volume. For both CGs, we then inoculated a biovolume of 2.5 × 10^9^ μm^3^ of each population into a 250 mL culture flask, filled up to 100 mL of media (biovolume density of 2.5 × 10^3^ μm^3^/μL). Both CGs lasted for 25 days until the samples reached carrying capacity. Each sampling day, we collected 10 mL from every culture flask for analysis (see details below) and replaced it with 10 mL of fresh media to maintain a constant volume. For both CGs, we measured on day 1, 2, 4, 5, 7, 9, 11, 14, 16, 18 (week 17 only), 21 and 25.

### 2.3 Cell Size, Densities and Biovolume

On each sampling day of the CG, we measured cell size (μm^3^), cell density (cells/μl) and biovolume (μm^3^/μL; calculated by multiplying cell density and average cell size for each replicate). Size and density were obtained from photos taken with light microscopy at 400× magnification (Olympus IX73 inverted microscope) after staining a 1 mL sample of each sample with 1% Lugol's iodine. Ten μL of the sample were loaded onto a Neubauer counting chamber (Marienfield, Germany) and 20 evenly spaced photographs were taken around the counting frame. All images were analysed using ImageJ and Fiji software (version 2.0) (Schindelin et al. 2012) to quantify the morphological characteristics (cell length and width) and cell densities. We then estimated cell volume by assigning either a prolate spheroid shape (*Amphidinium*, *Dunaliella*) or spherical shape (*Tisochrysis*) to each cell (Hillebrand et al. 1999).

### 2.4 Metabolic Rates

In conjunction with biovolume and density data, we measured photosynthesis and respiration rates to identify the physiological changes that underpin population‐level responses. For each lineage, we tracked changes in oxygen levels in 5 mL vials using established protocols (Ghedini and Marshall 2023). Prior to all experiments, the respirometry system (PreSens Sensor Dish Reader, SDR; PreSens Precision Sensing, Germany) was calibrated using 0% and 100% oxygenated seawater. To prevent carbon limitation, we added 50 μL of sodium bicarbonate stock (a final concentration of 2 mM sodium bicarbonate) to each sample vial before measurements began. The samples were measured for 20 min in the light to quantify photosynthesis, followed by 40 min of darkness to determine respiration rates. As the cultures were not axenic, sixteen blanks were filled with spent media with no phytoplankton cells to account for the background bacterial activity (spent media was obtained by centrifuging samples at 5000 rpm for 10 min to separate the algal cells from the supernatant). The photosynthesis and respiration rates (VO_2_; μmol O_2_/min) of each sample were calculated as:

$$VO_{2}=\frac{m_{a}-m_{b}}{100}\times V\beta O_{2}$$

where *m*_*a*_ is the rate of change in oxygen levels of the sample (min^−1^), *m*_*b*_ is the mean O_2_ level across all blank samples (min^−1^), *V* is the sample volume (0.005 L) and βO_2_ is the O_2_ capacity of air‐saturated seawater at 20°C and 35 ppt salinity (225 μmol O_2_/L) (White et al. 2011). The first 3 min of measurements under the light were discarded to allow acclimation; similarly, respiration rates were calculated after 20 min of dark when the O_2_ levels demonstrated a linear decline. Subsequently, we converted the photosynthetic and respiration rates (μmol O_2_/min) to calorific energy (J/min), using a conversion factor of 0.512 J/μmol O_2_ to estimate the energy production and consumption, respectively (Williams and Laurens 2010). These calculations were completed using the LoLinR package (Olito et al. 2017) in R (version 4.3.2) (R Core Team 2021).

### 2.5 Data Analysis

All statistical analyses were done on the data collected during the two common garden experiments (week 9, week 17; not during the evolution phase) to assess evolutionary differences instead of plastic responses. All data was analyzed using R (version 4.3.2) (R Core Team 2021) and Rstudio (R Studio Team 2021) using the packages nlme (Pinheiro and Bates 2022), lme4 (Bates et al. 2015), emmeans (Searle and Milliken 1980), car (Fox and Weisberg 2019), plyr (Wickham 2011a) for analyses and ggplot2 (Wickham 2011b; Wilke 2016) for creating plots. For all statistical analyses, interactions between the variables were removed from the model when *p* > 0.25. All figures with means show the least square means with a 95% confidence interval using a Tukey *p*‐value adjustment unless stated otherwise.

#### 2.5.1 Changes in *r* and *K* of Biovolume and Cell Densities

To determine how demography evolved over time (week 9, week 17) under each competition treatment (mono, poly), we quantified changes in the maximum rate of increase (*r*_max_) and the maximum value (carrying capacity, *K*) of both biovolume (μm^3^/μL) and cell density (cells/μL). We implemented the same approach used in (Malerba et al. 2018; Ghedini et al. 2022). Firstly, we fitted four growth models to the biovolume (or cell density) data of each replicate using non‐transformed data. The models were: a logistic‐type sinusoidal growth model with a lower asymptote forced to 0 (e.g., three‐parameter logistic curve), a logistic‐type sinusoidal growth model with a non‐zero lower asymptote (e.g., four‐parameter logistic curve), a Gompertz‐type sinusoidal growth model (e.g., three‐parameter Gompertz curve) and a modified Gompertz‐type sinusoidal growth model including population decline after reaching a maximum (e.g., four‐parameter Gompertz‐like curve including mortality). Secondly, we utilized the AIC (Akaike information criterion) to determine the best fitting models for each lineage with successful convergence, which we used to estimate the maximum predicted value (*K*) of biovolume or cell density for each replicate. From the first derivative, we extracted the maximum rate of increase (*r*_max_). Finally, we used a linear model to test for differences in *K* and *r*_max_ between species, experiments (week 9, week 17), and competition treatments (mono, poly). To avoid pseudoreplication for the mono treatment, the data from the two samples (bags) collected from the same beaker were averaged, resulting in *n* = 3 for the subsequent analyses (unless otherwise stated).

In the main text, we report the test on the percentage change of the population parameters (calculated from the difference in *K* or *r*_max_ at week 17 relative to week 9). For this analysis, we used a linear model that includes species identity (*Amphidinium*, *Dunaliella*, *Tisochrysis*) and competition treatment (mono, poly) as factors. We report the figures and analyses on differences in absolute values of *r*_max_ and *K* in the supplement (Figures S1 and S2), where we test for differences in population parameters of each species separately using a linear model that has initial biovolume (or cell density) as a covariate and competition treatment (mono, poly) and experiment (week 9, week 17) as factors, including their interactions.

From the best‐fitting growth model for each replicate, we also extracted the population growth rate (cells/μL) on each day of the common gardens (we did this on cell densities only because we also calculated per capita growth rates). We then analysed these growth rates from day 8 onwards (i.e., the declining phase) to determine changes in growth as species approached the stationary phase. We used a linear mixed effect model that includes growth rate as a response variable (log_10_‐transformed), CG Day, experiment (week 9, week 17), competition treatment (mono, poly) and species (including their interactions), and sample code as a random effect.

To visualise differences in growth trajectories (Figure S3), we fitted the same models described above to all replicates within a treatment (species, competition treatment and common garden experiment). Figure S3 shows the best‐fitting model based on AIC for each treatment combination.

#### 2.5.2 Changes in Density‐Dependence

To better understand the demographic changes in *r*_max_ and *K*, we fitted biovolume data with a logistic growth model with explicit *r*–*α* formulation (Adler et al. 2018). With this model, we can determine how the strength of intraspecific competition *α*_*ii*_ evolved over time as a function of competition type. We fit the model to biovolume data of each replicate (*B*_*i*_, μm^3^/μL) because biovolume is the quantity that is more comparable and evolves in the same way among species:

(3) $$\frac{dB_{i}}{\mathrm{dt}}=rB_{i}\left(1-\alpha_{\mathrm{ii}}B_{i}\right)$$

Note that $\alpha_{\mathrm{ii}}=1/K$ in Equation (3) while α=r/K in Equation (2) (so $\alpha_{\mathrm{ii}}=\alpha /r$) but both models (Equations 2 and 3) recover the *r*–*K* formulation in Equation (1); we use Equation (3) because we explore the relationship between $\alpha_{\mathrm{ii}}$ and *r*. To do that, we used a linear model to determine how *α*_*ii*_ changes as a function of intrinsic rate of increase (*r*), experiment (week 9, week 17), competition treatment, and species. Interactions were removed when *p* > 0.25 so the final model took the form of: α_*ii*_ ~ *r* + experiment + treatment + species + *r* × species + experiment × treatment + experiment × species + treatment × species. For this analysis we used all replicates (not averaging between beakers) because we were interested in the evolution of density‐dependence and its relationship with *r* within each population. We excluded one *Amphidinium* sample from the polyculture treatment that had an *α*_*ii*_ three times that of all other *Amphidinium* samples (3.6e‐06 vs. ~1.2e‐06), due to unusually slower growth in the first days of the common garden.

#### 2.5.3 Metabolic Rates

To explore the physiological basis of the demographic responses, we tested for changes in net energy production (per cell) with respect to population biovolume. First, we estimated per capita rates of photosynthesis or respiration by dividing the population rate of each replicate by its total population size (cells in 5 mL). We then estimated per capita net energy production over a 24‐h period (J/day/cell) as 12 h of energy produced through net photosynthesis minus 12 h of respiration. We removed day 0 (as no oxygen rates were measured on that day), as well as day 1 and day 2 because many respiration rates were negative (the biovolume was low so respiration rates were often not different or lower than blanks). We excluded an additional 18 data points that had negative values (16 for respiration rates and 2 for net energy). This left us with 713 data points in total. On this dataset, we fitted a linear mixed‐effect model with per capita net energy production (log_10_‐transformed) as a response variable, population biovolume (log_10_‐transformed) as a covariate, experiment, competition treatment, and species as factors, and sample code as a random effect.

To understand what was driving the change in net energy, we compare per capita photosynthesis and respiration rates in the exponential (day 4) and stationary phase (day 25). We use a simple linear model with per capita rates as the response variable (log_10_‐transformed); species identity (*Amphidinium*, *Dunaliella*, *Tisochrysis*), ‘condition’ and growth phase (exponential vs. stationary) as predictors, including their interactions. Note that here ‘condition’ combines both Experiment and competition treatment (i.e., week9_mono, week9_poly, week17_mono, week17_poly) to avoid overfitting the model (and since we have already seen from the analysis above that the type of competition had small effects on net energy per capita).

#### 2.5.4 Changes in Cell Size

To determine how cell size evolved, we compared cell sizes on day 0 of each common garden with the ancestral cell size of each species. We used a linear model with the cell size (log_10_‐transformed to meet normality assumptions) as the response variable, species (*Amphidinium*, *Dunaliella*, *Tisochrysis*) and condition (ancestor, week9_mono, week9_poly, week17_mono, week17_poly) as factors, including their interactions. Then we explored changes in size as populations grew during the common garden experiments for each species separately since they show different trajectories. We used a linear model that included competition treatment, common garden experiment, and common garden day as factors, including their interactions.

## 3 Results

### 3.1 All Species Increase Population Production in Response to Intra‐ and Inter‐Specific Competition as Predicted by Life History Theory

**FIGURE 2:**
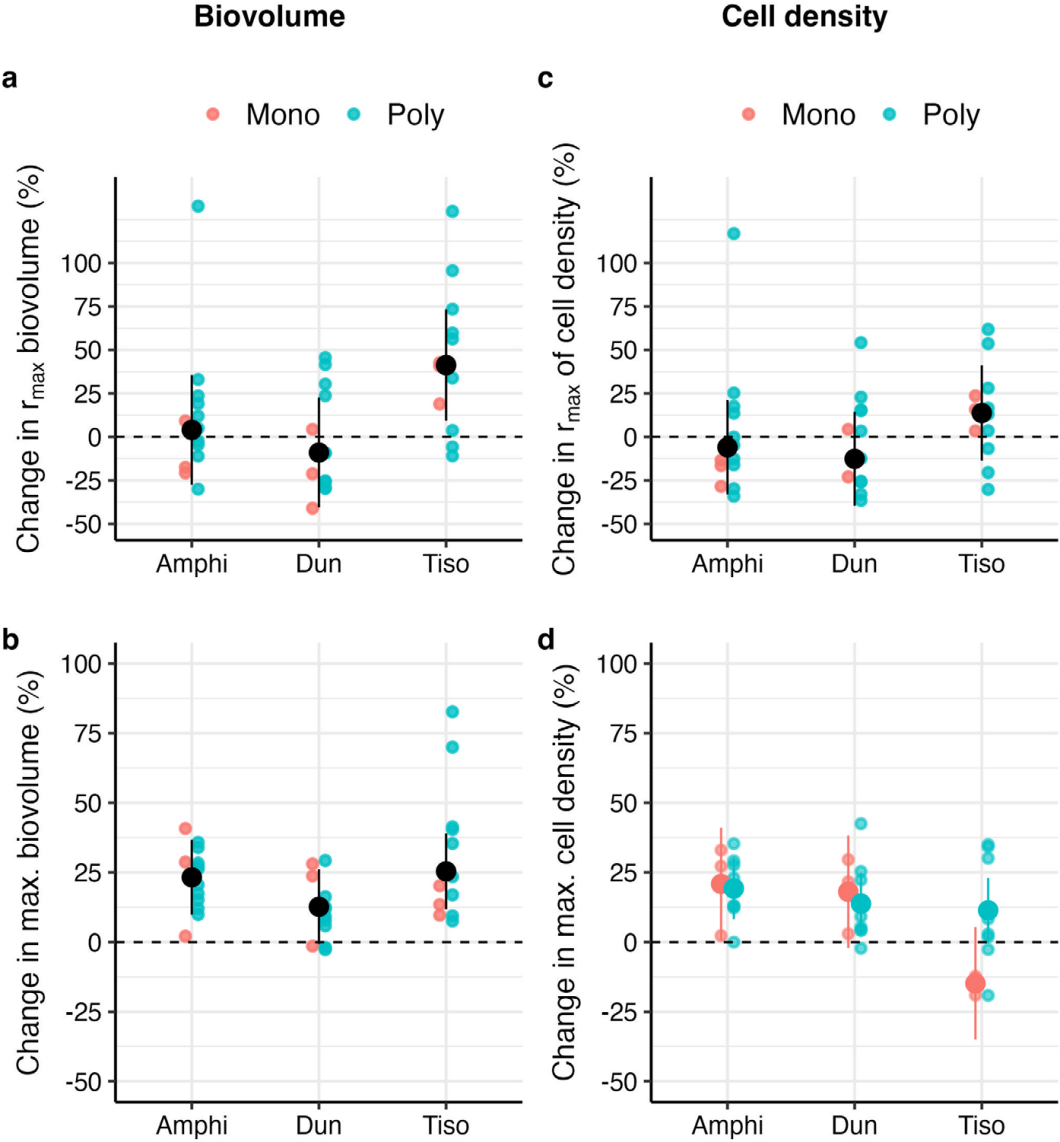
Percentage change in the maximum rates of increase (r_max_) and maximum values (*K*) of biovolume (a, b) and cell density (c, d) from the first to the second common garden (positive values indicate an increase, negative values a decrease from week 9 to week 17). All species increased the maximum biovolume (b) and two out of three also increased their cell density (d). *Tisochrysis* showed different changes in cell density depending on the competition treatment. Maximum growth rates did not change, either for biovolume (a) or cell density (c), except for *Tisochrysis* that evolves faster max. rates of biovolume production. Refer to Table S1 for the model outputs.

Our results validate the predictions of life history theory, as all three species evolved greater population production at week 17 relative to week 9. This increased production was driven by an increase in biovolume carrying capacity (*K*) at no expense of max. growth rates (r_max_) (Figure 2a,b; Figures S1–S3). Max. biovolume increased by approximately 20%, and the strength of this response was not affected by the type of competition (intra‐ or inter‐specific) (Tables S1 and S2). *Tisochrysis* was the only species to show a change in the max. rate of biovolume production (*r*_max_) which increased from week 9 to week 17, and more so in the polyculture treatment (Figure 2a, Figure S1, Table S2).

In two out of the three species (*Amphidinium*, *Dunaliella*), biovolume carrying capacity increased because populations sustained more cells (higher *K* in cell number) (Figure 2d; Figure S2; Table S3). In the third species (*Tisochrysis*) instead, biovolume increased because cells were larger (Figure S4). Max. growth rates (number of cells) did not change for any of the species or treatments (Figure 2c, Figure S2).

In general, all three species evolved smaller cells in comparison to their ancestors, at least when considering the size at the start of common garden experiments (Figure S5; Table S4). But these changes in size were not always maintained during the common garden experiments, and there were no clear differences between competition treatments (Figure S4, Table S5).

### 3.2 The Evolution of Reduced Density‐Dependence Explains Increased Production

To better understand why species increased their biovolume production, we assessed changes in the density‐dependence of biovolume growth (α_*ii*_; describes the sensitivity to intraspecific competition) and net energy per capita.

**FIGURE 3:**
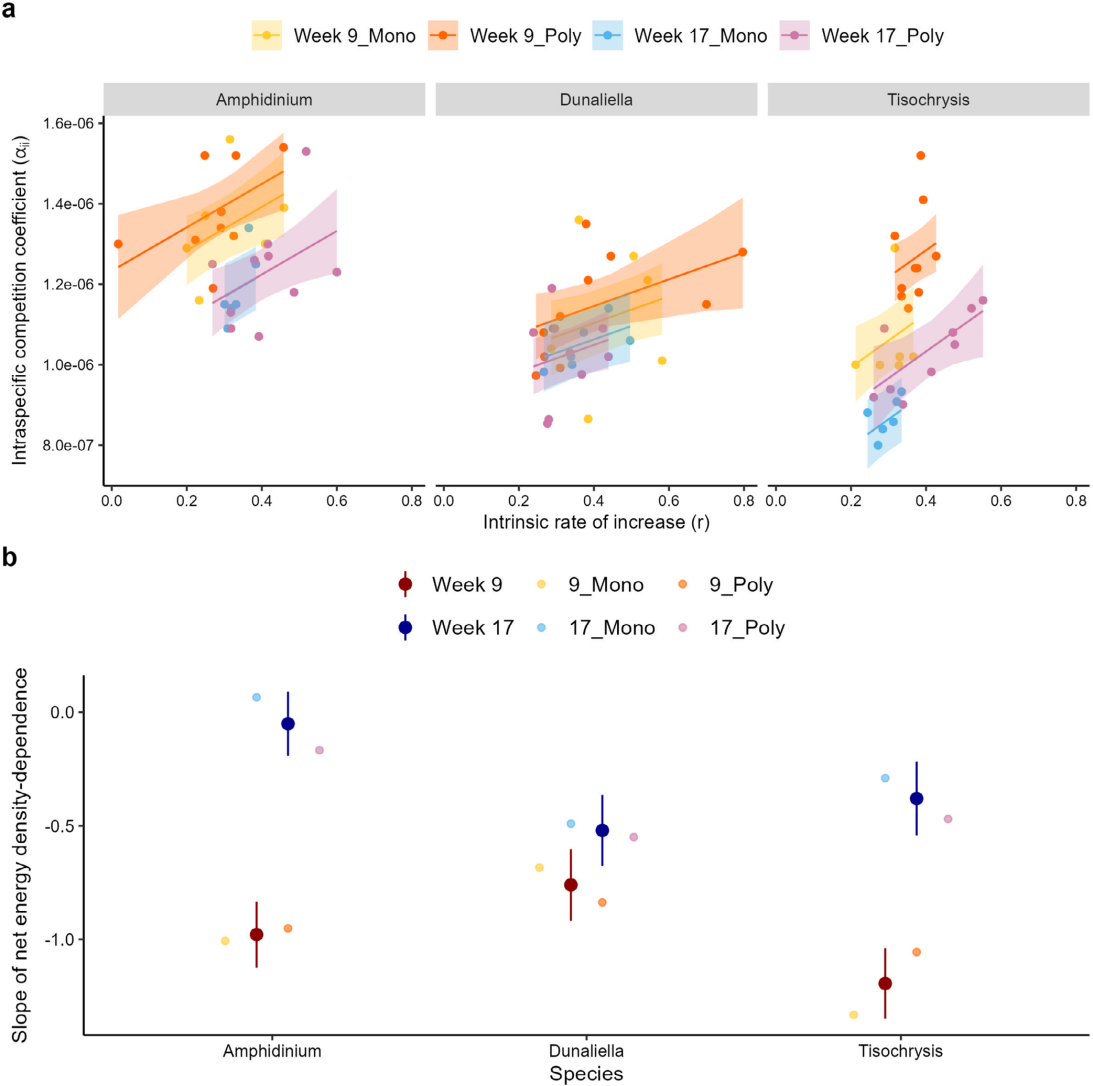
(a) The strength of intraspecific density‐dependence (α_*ii*_ calculated on biovolume data) declines over time (from week 9—warm colours—to week 17—cold colours) across all species, although this decline is not significant for *Dunaliella* (posthoc comparison between week 9 and week 17: *p* = 0.09 for *Dunaliella*). Independently of time, density‐dependence was stronger in populations from polycultures tha in monocultures (Table S6). (b) Simultaneously, all species evolved a weaker density‐dependence of net energy production at week 17 (compared to week 9). The plot shows the value of the slopes (±95% confidence intervals) of the relationship between net energy per cell and total biovolume; slopes were shallower in week 17 compared to week 9. At week 17, slopes also tended to be more negative for species evolved in polyculture, but this effect was marginal (Table S7; for simplicity we only show the mean slope, without CI, for the competition treatments).

We analysed α_*ii*_ as a function of intrinsic rate of increase (*r*) since both parameters influence *K*. There was no change in the relationship between α_*ii*_ and *r* across species or treatments (slopes were the same, Figure 3a, Table S6). However, the parameter α_*ii*_ declined from week 9 to week 17 across the entire range of *r* (experiment × species: *F*_2,81_ = 4.66, *p* = 0.012; Figure 3a; Table S6). This decline was significant for *Amphidinium* and *Tisochrysis* (*p* < 0.0001), but marginal for *Dunaliella* (*p* = 0.0948). A smaller α_*ii*_ indicates a lower sensitivity to intraspecific competition: growth declines less rapidly with total biovolume; thus all three species evolved a lower density‐dependence of biovolume growth. On average, the populations that evolved with interspecific competitors (polyculture) had higher α_*ii*_ (stronger density‐dependence) than those evolved in monoculture (competition effect: *F*_1,81_ = 8.89, *p* = 0.004; Table S6).

**FIGURE 4:**
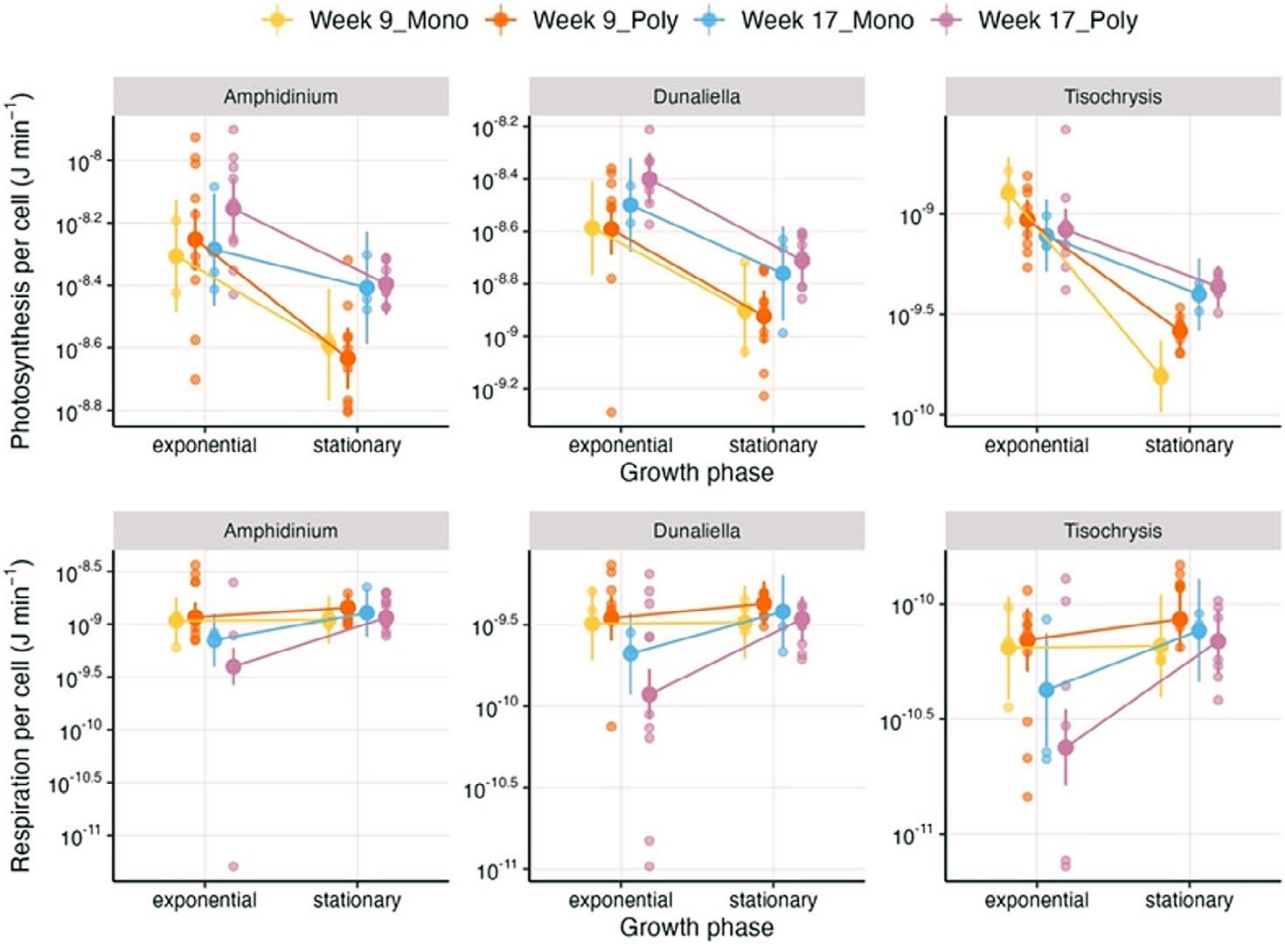
Reaction norms showing changes in per capita photosynthesis (top row) and respiration (bottom row) from exponential (day 4) to stationary phase (day 25) for the two common gardens and competition treatments. All species show an increase in photosynthesis rates from week 9 (warm colours) to week 17 (cold colours) in stationary phase. The strength of this increase varies between species and treatments (for *Amphidinium* and *Dunaliella* it is significant only in the polyculture treatment). Changes in the exponential phase are not significant, except for *Dunaliella* between week 9 and week 17 in the polyculture treatment. Conversely, there are no changes in respiration rates in the stationary phase, but respiration rates are lower at week 17 compared to week 9 in the exponential phase. Refer to Table S8 for the model outputs.

Changes in the density‐dependence of biovolume growth occurred in parallel to changes in energy fluxes. The slopes relating per capita net energy to total biovolume (log–log transformed) were shallower at week 17 compared to week 9 for all species (Figure 3b; Table S7). This meant that, after evolving with competitors for 17 weeks, cells produced more net energy in dense populations (Figure S6). The evolution of net energy was marginally affected by the type of competition: density‐dependence at week 17 tended to be stronger for populations evolved with interspecific competitors (polyculture; Figure 3b; log_10_(biovolume) × experiment × competition treatment interaction: *F*_1,645_ = 2.9, *p* = 0.08; Table S7), in agreement with their higher α_*ii*_ (Table S6). Increases in net energy were primarily driven by changes in photosynthesis more than respiration; per capita photosynthetic rates were higher at week 17 compared to week 9, particularly in the stationary phase (Figure 4, Table S8).

### 3.3 Increased Net Energy Fluxes Sustain Faster Growth in Competitive Environments

Ultimately, changes in density‐dependence and net energy translated into more population growth when competition was intense. Population (and per capita) growth rates declined less steeply as populations approached carrying capacity in week 17 compared to week 9 (Figure S7; Table S9). The only exception was *Tisochrysis* evolved in monoculture, which showed the opposite pattern, since here we are looking at cell densities (these populations achieved greater biovolume through changes in cell size, not cell densities). Again, populations evolved with interspecific competitors (polyculture) were less ‘fit’ than populations evolved in monoculture because their growth rates declined faster (Figure S7b, Table S9), consistent with the negative effects of interspecific competition on density‐dependence (α_*ii*_) and net energy.

## 4 Discussion

Life history theory predicts that competition should increase population production (Lande et al. 2009; White and Marshall 2023), but there are few empirical tests under interspecific competition. Therefore, the generality of this prediction remains unclear. MacArthur and Wilson (Macarthur and Wilson 1967) hypothesized that, in a community of species that compete for similar resources, interspecific competitors should compound the effects of intraspecific competition, leading to similar evolutionary outcomes—which should be particularly true when species compete for essential resources. Our results validate these predictions: all three species tested evolved greater population production in response to intra‐ and interspecific competition, increasing biovolume carrying capacity (*K*) at no expense of max. growth rates (*r*_max_). Our species achieved this same outcome in two different ways, either by evolving greater maximum cell densities or larger cell sizes (Figure 1b). This result suggests that evolution in response to competition leads to trait changes that increase population production in the form of biovolume; it further suggests that different species can maximize production through different strategies. We can speculate that smaller species (e.g., *Tisochrysis*, the smallest species we tested) might be more likely to increase biovolume through the evolution of larger cell sizes (rather than densities), but this can only be confirmed with further studies incorporating more species.

Not only did competition lead to the same consistent outcome across species and competition treatments (i.e., greater total biovolume), but we also found that the underlying physiological mechanisms were the same. Evolution reduced density‐dependence (α_*ii*_) so that cells sustained higher net energy fluxes and growth rates in crowded environments (closer to carrying capacity, when competition was highest). Net energy results from the balance of photosynthesis and respiration over 24 h. Therefore, net energy increases either by reducing respiration costs or increasing energy production through photosynthesis. We found that the latter was the main driver in our experiment, because photosynthetic rates were upregulated across all species in the stationary phase after evolution. While we did not further explore how photosynthesis increased, cells can enhance productivity through chlorophyll synthesis and packing (Malerba et al. 2018). Processes of energy intake/production and expenditure can thus respond differently to competition, not only in terms of plastic changes (Ghedini et al. 2017) but also evolutionary responses (Marshall et al. 2022). This differential regulation of energy fluxes might be very important for fitness because organisms can increase energy gains without increasing metabolic costs.

While we cannot establish if physiology drives demography or the other way around, net energy and competitive ability improved in parallel. Reduced sensitivity to intraspecific competition (α_*ii*_) evolved as a strategy to minimize competition from both intraspecific and interspecific competitors: species evolved in monoculture and polyculture all had lower α_*ii*_ at the end of the experiment (Figure 3a), similarly to what has been observed in plants (Sakarchi and Germain 2023). Improvements in competitive ability did not come at the cost of maximum growth rates; on the contrary max. growth rates improved in one species. Trade‐offs between traits, such as *r*_max_ and *K*, are not always observed even though they are often predicted (Marshall et al. 2023); if anything, *r*_max_ and *K* should be positively correlated (Mallet 2012; Marshall et al. 2023; Wei and Zhang 2019). While we do not find much evidence that *r*_max_ evolves, we clearly observed that growth rates improved when populations were dense (allowing them to accumulate more biovolume and increase *K*). This result echoes the changes in growth rates observed in flies: fly populations evolved in crowded conditions have a higher rate of per capita growth at high densities, but these improvements in performance do not manifest at low densities (Muellert and Ayala 1981). Overall, it seems that competition maximizes population production through changes in energy use that increase energy gains and the conversion of this energy into biomass (i.e., increasing growth when competition is intense) (Macarthur and Wilson 1967; Marshall et al. 2022).

Why do max. growth rates not evolve? The adaptation of organisms to ‘harsh’ environments (such as intense competition) should result in a trade‐up of *r*_max_ and *K* if traits are far from optimised (Sakarchi and Germain 2023; Lande et al. 2009; White and Marshall 2023; Engen and Sæther 2016, 2017; Marshall et al. 2023), as should be in our experiment since none of the species experienced this experimental setup before. Similarly, variations in selective pressures due to environmental fluctuations (e.g., transfers from low to high densities as in our batch transfer approach) should optimise both max. growth rates (i.e., growth when resources are abundant) and competitive ability (i.e., efficiency when resources are scarce) (Macarthur and Wilson 1967; Lande et al. 2009; Holdridge and Vasseur 2022). Instead, only one species (*Tisochrysis*) improved both *r*_max_ and *K*. Since the strength of selection is linked to environmental conditions (Wei and Zhang 2019), stronger fluctuations between low‐ and high‐density environments (e.g., more frequent or larger dilutions) might increase selection pressure and result in the evolution of r_max_ across more species (Hart et al. 2019).

As predicted by MacArthur, evolutionary responses were very similar in response to both intra‐ and interspecific competition. This similarity could be due to an experimental limitation: dialysis bags prevent species from being in direct contact, which can increase intraspecific competition at the expense of interspecific competition in the polyculture treatment. This dialysis bag approach was, however, essential to phenotype each species independently in common gardens (cell sorting was not a viable approach because population recovery and growth was extremely slow). Nonetheless, when species compete for essential resources, the identity of the competitor might be of little importance (Macarthur and Wilson 1967; Abrams 1986). For instance, the metabolism and growth of phytoplankton seem primarily affected by the total amount of competition (determined by total biovolume) and less so by the specific composition of biovolume (i.e., the abundance of intra‐ and inter‐specific competitors) (Fant and Ghedini 2024).

Despite the overall similarity in evolutionary responses, we observed consistent differences in the strength of responses to intra‐ and interspecific competition. Reductions in density‐dependence were weaker when populations evolved in polyculture: these populations had higher intraspecific competition coefficients (α_*ii*_; Figure 3a), stronger density‐dependence of net energy (Figure 3b), and reduced population growth compared to those evolved in monoculture (Figure S7). These effects were generally small and did not affect the main evolutionary outcome (increased biovolume production). But they show that the presence of interspecific competitors weakened evolution; this might happen in two opposing ways (Osmond and de Mazancourt 2013). The presence of interspecific competitors might allow resource partitioning between species (Striebel et al. 2009), thus reducing the selective pressure of competition relative to monoculture treatments (if total biovolume is the same)—which could explain why density‐dependence evolved ‘less’ for populations in polyculture. Alternatively, if resources are more fully utilised in communities, species in polyculture might experience stronger resource limitation and fewer opportunities to evolve niche diversification (which is a way to alleviate density‐dependence and improve growth) (Scheuerl et al. 2020; De Mazancourt et al. 2008; Svanbäck and Bolnick 2007). The reduced fitness of species evolved in polyculture (i.e., faster decline in growth rates as populations approach stationary phase, Figure S7) would point to this latter explanation.

All three species increased population biovolume regardless of their size, even though phytoplankton cell size affects metabolic rates, resource uptake, and competitive ability (Litchman et al. 2007). However, the three species we tested maximized biovolume through different life history strategies (by increasing max. population size or individual size), although we cannot establish if these strategies are driven by differences in size. We initially included a fourth species, *Nannochloropsis* (the smallest species), which went extinct halfway through the evolution experiment and first in the polyculture treatment. Smaller algae have a higher surface‐to‐volume ratio, allowing for greater rates of resource uptake per unit of biomass and suffering less from self‐shading (Gallego and Narwani 2022); therefore, they should be strong competitors. However, small cells have lower storage capacity and poorer recovery from nutrient depletion (Malerba et al. 2018, 2020). The advantage of larger cells in terms of nutrient storage could explain why *Nannochloropsis* was competitively excluded and why *Tisochrysis* (the second smallest species) increased its size in response to competition (Hillebrand et al. 2022). The other two species reduced size in comparison to the ancestor (similarly to Ghedini and Marshall 2023), but these species had larger cell volumes (Fant and Ghedini 2024). Future experiments using species with greater size differences could clarify whether cell size influences evolutionary responses to competition.

## 5 Conclusions

In summary, our results validate the prediction of life history theory that evolution in response to competition reduces density‐dependence and maximizes population production (Macarthur and Wilson 1967; Abrams 1986). Our work shows that intra‐ and interspecific competition lead to the same evolutionary outcomes in phytoplankton, suggesting that similar patterns might hold when species compete for essential resources. We further show the common physiological mechanism that underpins these demographic responses and its connection with competitive ability (Figure 1b). The differential evolution of photosynthesis and respiration weakened the density‐dependence of net energy fluxes—therefore, cells produced more net energy (and thus biomass) in crowded conditions. These physiological changes also meant that the sensitivity to intraspecific competition declined (α_*ii*_) and growth rate increased. The concomitant assessment of the physiological and demographic traits that underpin density‐dependence can clarify the mechanisms that drive evolution. Determining these eco‐evolutionary dynamics seems particularly important in the face of rapid biodiversity and environmental changes (Loreau et al. 2023).

## Author Contributions

**Charlotte L. Briddon:** data curation (lead), formal analysis (equal), investigation (equal), methodology (equal), project administration (equal), resources (equal), software (equal), writing – original draft (lead), writing – review and editing (equal). **Ricardo Estevens:** data curation (equal), methodology (equal), resources (equal), writing – review and editing (equal). **Giulia Ghedini:** conceptualization (lead), formal analysis (equal), funding acquisition (lead), investigation (equal), project administration (equal), supervision (lead), validation (equal), visualization (equal), writing – review and editing (equal).

## Conflicts of Interest

The authors declare no conflicts of interest.

## Supporting information


Data S1.

## Data Availability

All data and code have been deposited in Figshare and can be accessed here: https://doi.org/10.6084/m9.figshare.27102136.v1.
