# Supplementary material for: Evolution Under Competition Increases Population Production by Reducing the Density‐Dependence of Net Energy Fluxes and Growth
Source: Ecol Evol. 2025 Mar 17;15(3):e71071. doi: 10.1002/ece3.71071 (PMC11913549; doi:10.1002/ece3.71071)
Supplement: Supplementary file 1 — Data S1. [file ECE3-15-e71071-s001.docx]

**Supplementary Information**

**Evolution under competition increases population production by reducing the density-dependence of net energy fluxes and growth**

Charlotte L. Briddon^1*^, Ricardo Estevens^1^, Giulia Ghedini^1,2*^

^1^ GIMM - Gulbenkian Institute (previously Instituto Gulbenkian de Ciência), Lisbon, Portugal

^2^ School of Biological Sciences, Monash University, Clayton 3800, Victoria, Australia

*Joint corresponding authors: charlybriddon1@gmail.com and giulia.ghedini@monash.edu


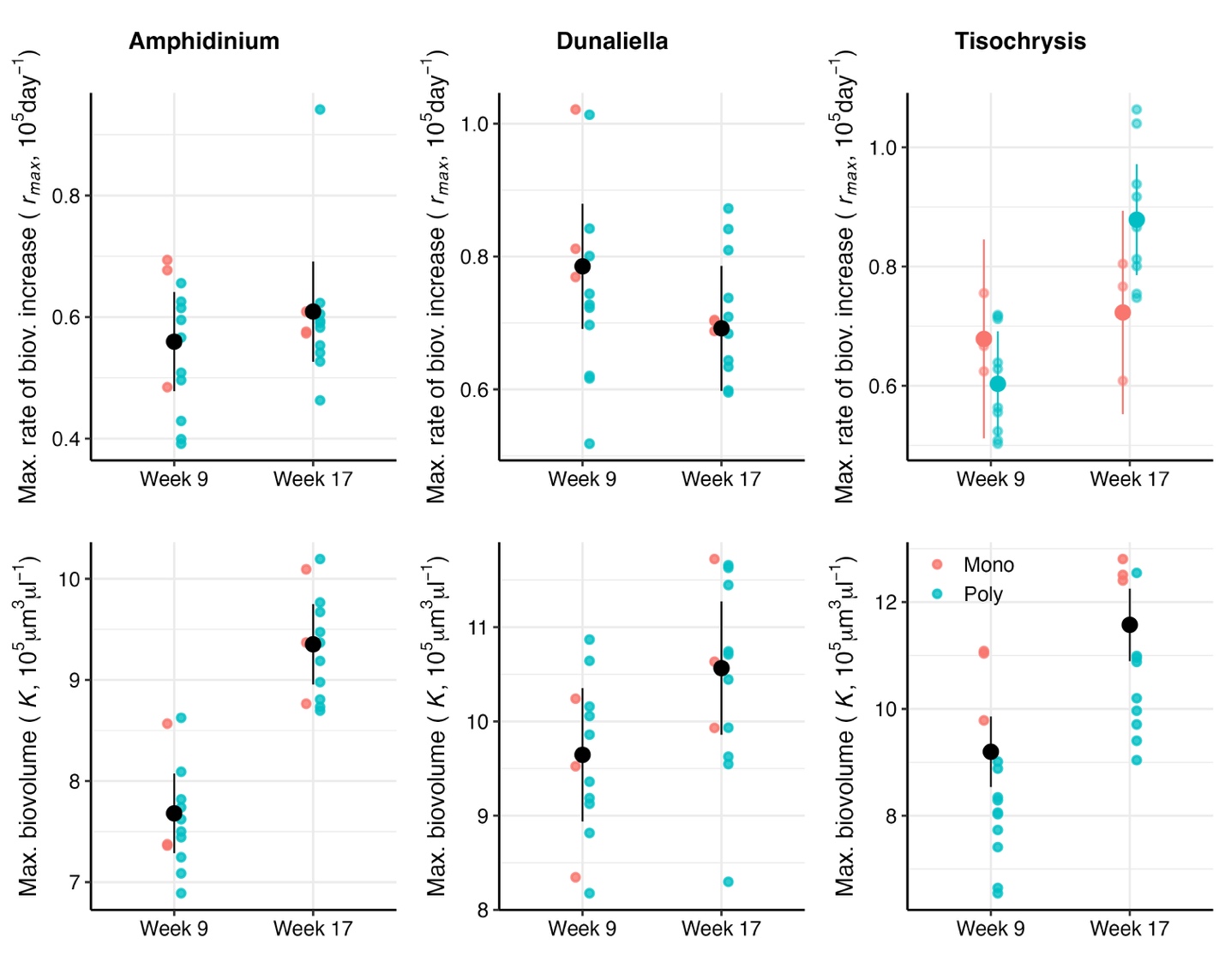


**Figure S1.** Change in maximum rate of increase (r_max_) and max value (K) of biovolume (μm^3^/μl) for each species after 9 and 17 weeks of evolution alone (monoculture) or in a community (polyculture). Refer to Table S2 for the model outputs.


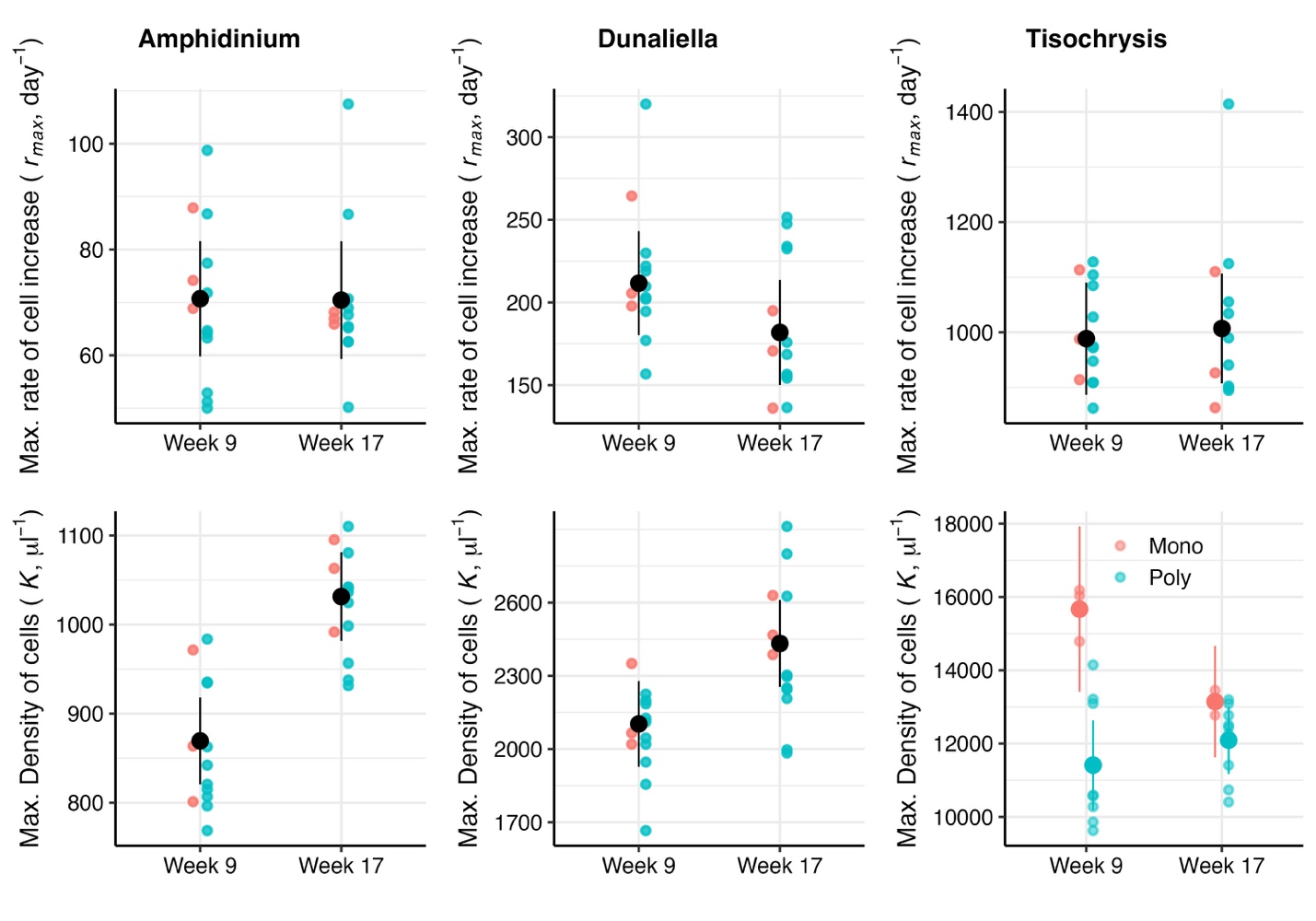


**Figure S2.** Change in maximum rate of increase (r_max_) and max. population density (K) quantified as number of cells (cells/µl) for each species after 9 and 17 weeks of evolution alone (monoculture) or in a community (polyculture). Refer to Table S3 for the model outputs.


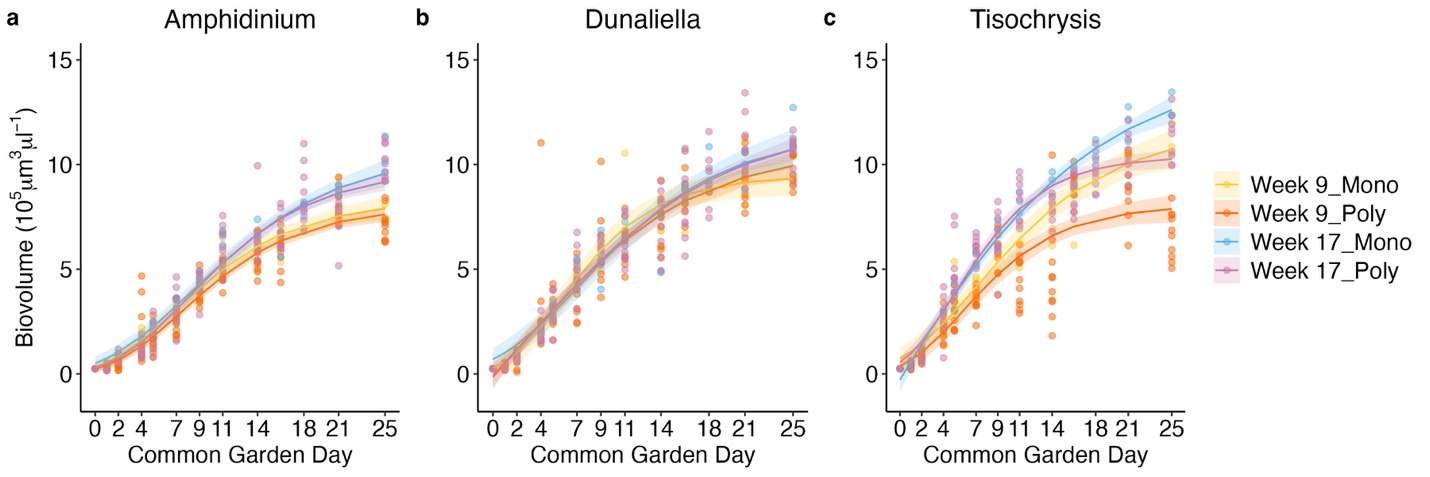


**Figure S3.** Biovolume carrying capacity increases over time across all species. These increases are similar between species evolved under intra- (monoculture) or inter-specific competition(polyculture), except for *Tisochrysis* (which populations have higher biovolumes when evolved in monoculture compared to polyculture at both timepoints).

**
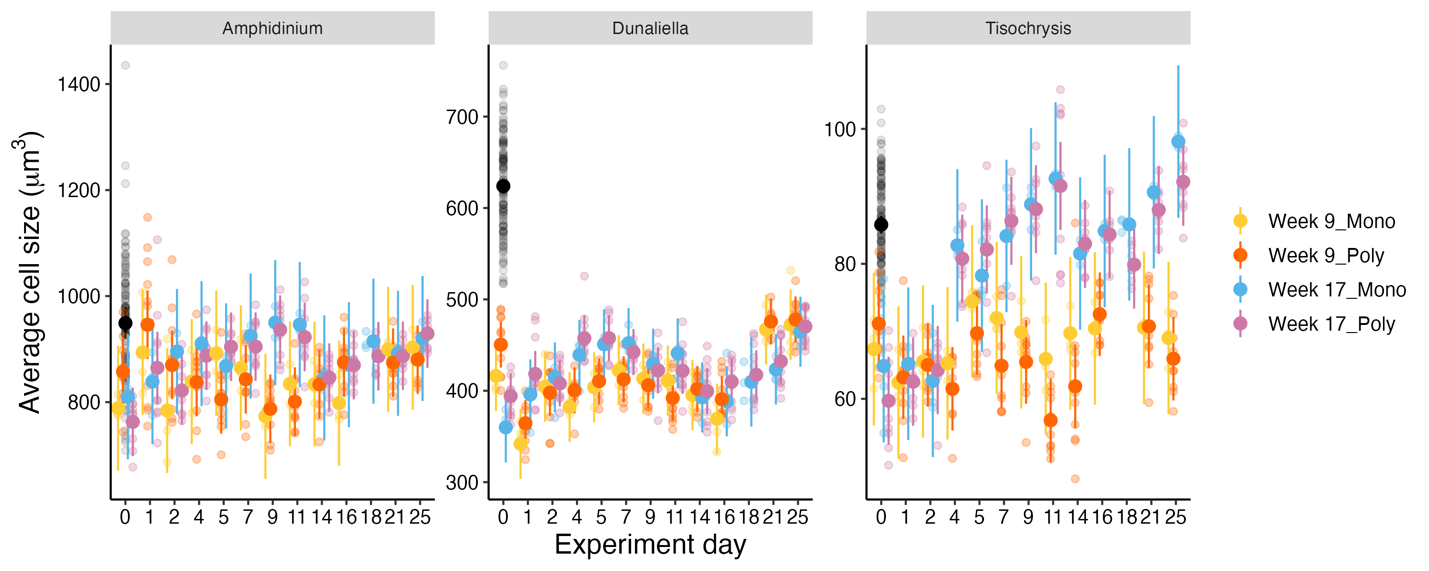
Figure S4.** The figure shows the cell sizes (the average cell size of each replicate) and the estimated marginal means with lower and upper 95% confidence intervals on each day of the common garden experiments (week 9 and week 17). We also include the mean cell size of the ancestors (measured on the first day of the evolution phase) for comparison (black). While all species reduce their cell size relative to the ancestors, these changes are not always maintained during the common garden experiments as cell size tends to increase (depending on the species). This increase is particularly strong for *Tisochrysis* in the second common garden (week 17) and explains why this species increases max. biovolume without increasing its cell density. Refer to Table S5 for the model outputs.


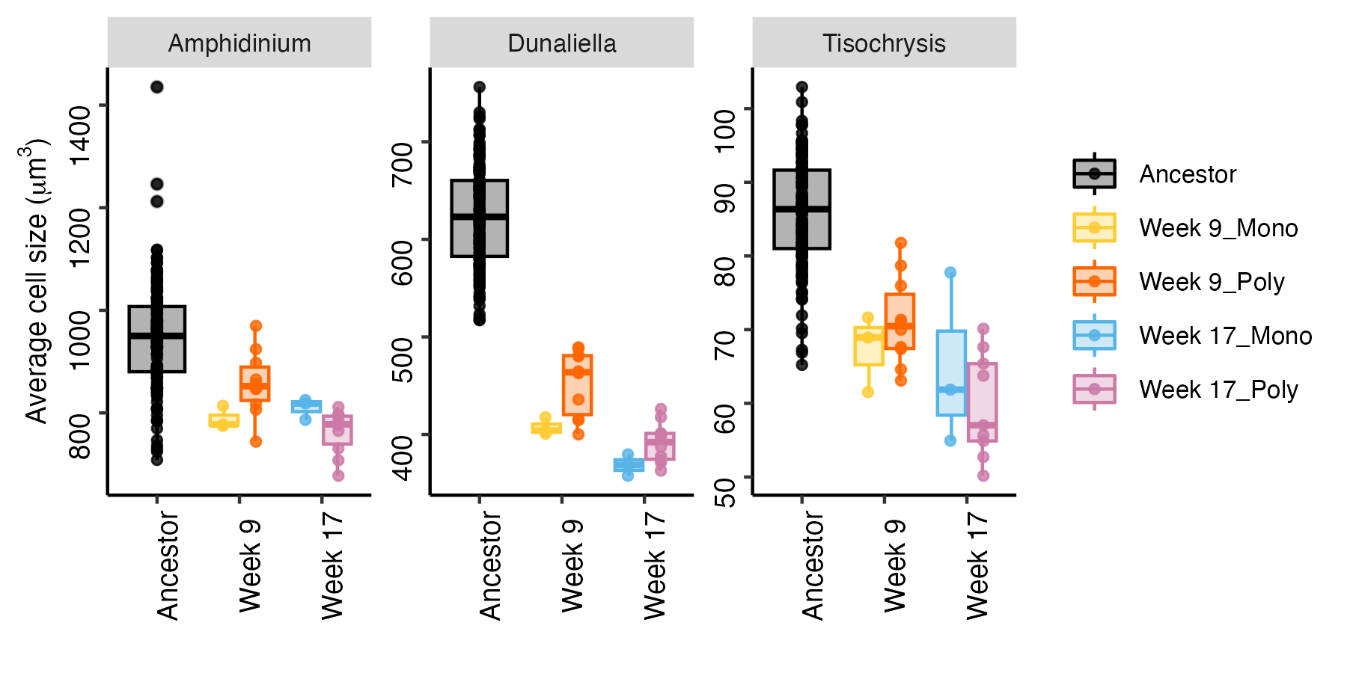


**Figure S5.** All species had smaller cells at the start of common garden experiments (i.e. day 0, after four days of neutral selection) in comparison to their ancestors. We find weak changes in size for each species between the two common garden experiments (week 9 and week 17) and competition treatments (mono vs poly). Refer to Table S4 for the model outputs.


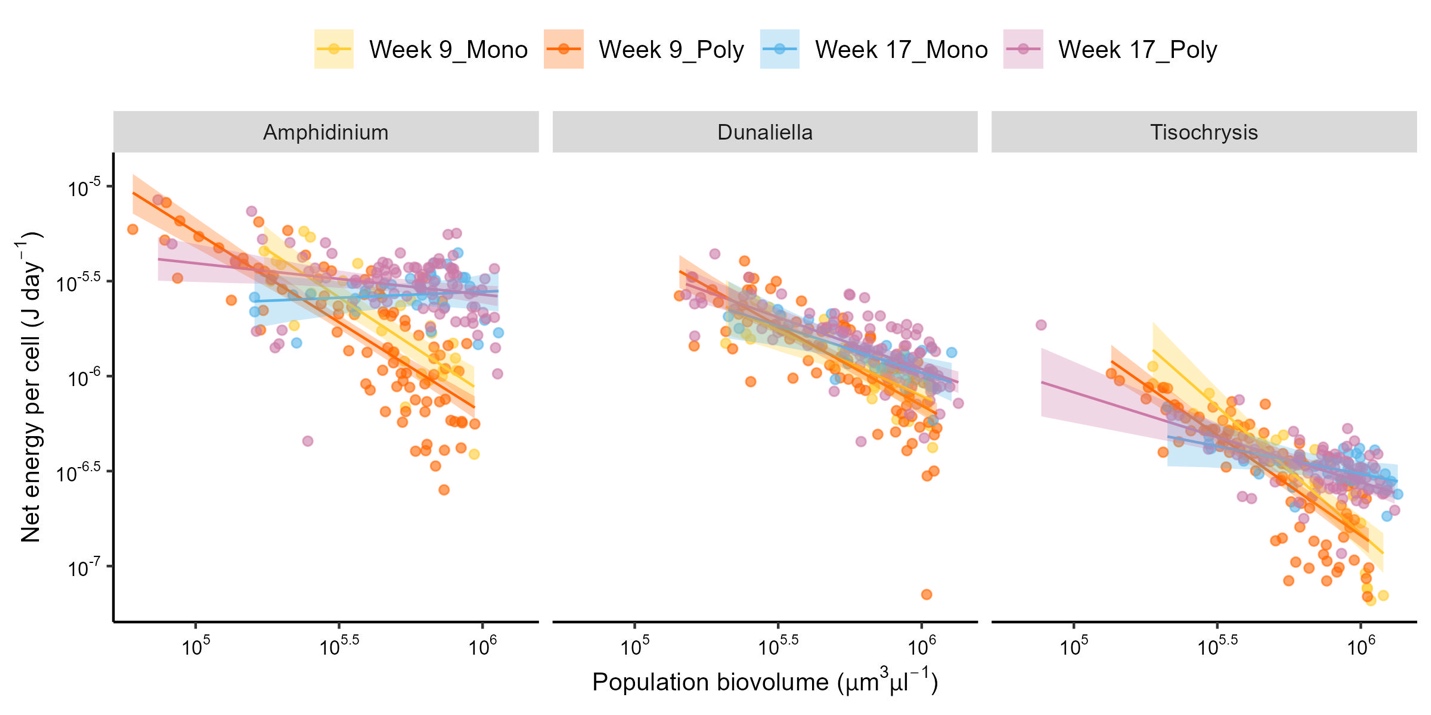


**Figure S6.** Over time, all species evolved a weaker density dependence of *per capita* net energy production (slopes were shallower in week 17 compared to week 9; log_10_(biov) × Experiment × Species: F_2, 645_ = 11.16, p < 0.001). Across all species, this reduction in density-dependence was stronger for populations evolved in monoculture in comparison to those evolved in polyculture but this difference was not significant (p = 0.09). Refer to Table S7 for the slopes and model outputs.

**
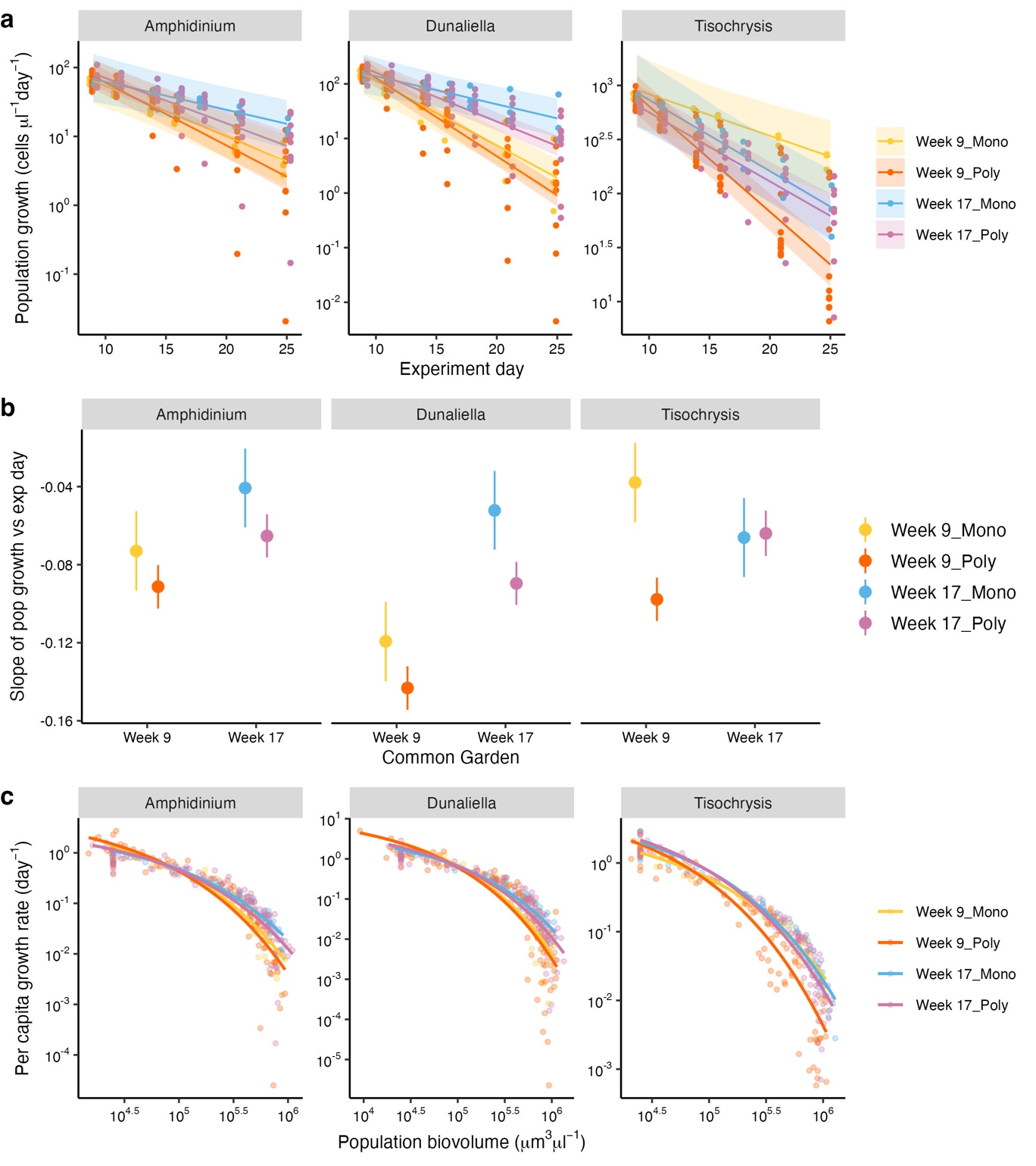
**

**Figure S7.** a) Population growth rate (cells μl^-1^ day^-1^) declines as species approach stationary phase (x-axis shows experiment day of common gardens), but this decline is shallower in week 17 (blue and purple) compared to week 9 (yellow and orange) – with the exception of *Tisochrysis* in the monoculture treatment (which increased biovolume through changes in cell size instead of changes in densities). Within each common garden, populations evolved in polyculture tend to have stronger density-dependence than those evolved in monoculture. Panel (b) shows the slope and 95% confidence intervals of this relationship. Refer to Table S9 for the model outputs. c) *Per capita* growth rates as a function of population biovolume show the same pattern.

| **Table S1**: Linear models and post-hoc test results showing the effect of competition treatment and species on the percentage change in max. growth rates (r_max_) and max. values (K) of biovolume (µm^3^/µl) or cell density (cells/µl). Relates to Figure 2. P values < 0.05 are in bold. CL = 95% confidence level. Species abbreviations are: *Dunaliella* (Dun), *Amphidinium* (Amphi) and *Tisochrysis* (Tiso). Competition treatments are mono = monoculture, poly = polyculture. | | | | | |
| --- | --- | --- | --- | --- | --- |
| Percentage change in the max rates of increase of biovolume (Figure 1a) | | | | | |
|  | Df | Sum Sq | Mean Sq | F value | Pr(>F) |
| Species | 2 | 15157 | 7578.5 | 5.5275 | **0.008346** |
| Treatment | 1 | 2975 | 2975.4 | 2.1702 | 0.149909 |
| Residuals | 34 | 46616 | 1371.0 |  |  |
|  |  |  |  |  |  |
| Estimated Marginal Means | Estimate | SE | Lower CL | Upper CL |  |
| Dun | -9.08 | 11.0 | -36.6 | 18.4 |  |
| Amphi | 5.82 | 11.0 | -21.7 | 33.3 |  |
| Tiso | 39.64 | 11.3 | 11.4 | 67.9 |  |
|  |  |  |  |  |  |
| Contrast | Estimate | SE | Df | t ratio | p value |
| Amphi - Dun | 14.9 | 14.5 | 34 | 1.026 | 0.5659 |
| Amphi - Tiso | -33.8 | 14.8 | 34 | -2.281 | 0.0722 |
| Dun - Tiso | -48.7 | 14.8 | 34 | -3.286 | **0.0065** |
|  |  |  |  |  |  |
|  |  |  |  |  |  |
| Percentage change in max. values of biovolume (Figure 1b) | | | | | |
|  | Df | Sum Sq | Mean Sq | F value | Pr(>F) |
| Species | 2 | 2656.3 | 1328.15 | 5.074 | **0.01221** |
| Treatment | 1 | 110.5 | 110.54 | 0.4221 | 0.52053 |
| Species × treatment | 2 | 1130.2 | 565.12 | 2.1578 | 0.13210 |
| Residuals | 32 | 8380.5 | 261.89 |  |  |
|  |  |  |  |  |  |
| Estimated Marginal Means | Estimate | SE | Lower CL | Upper CL |  |
| Dun | 12.7 | 5.33 | -0.72 | 26.1 |  |
| Amphi | 23.3 | 5.33 | 9.84 | 36.7 |  |
| Tiso | 25.4 | 5.33 | 11.81 | 39.0 |  |
|  |  |  |  |  |  |
| Contrast | Estimate | SE | Df | t ratio | p value |
| Amphi - Dun | 10.56 | 7.53 | 32 | 1.402 | 0.3517 |
| Amphi - Tiso | -2.14 | 7.58 | 32 | -0.282 | 0.9571 |
| Dun - Tiso | -12.70 | 7.58 | 32 | -1.676 | 0.2299 |
|  |  |  |  |  |  |
| Percentage change in the max rates of increase of cell density (Figure 1c) | | | | | |
|  | Df | Sum Sq | Mean Sq | F value | Pr(>F) |
| Species | 2 | 2657 | 1328.7 | 1.2882 | 0.2889 |
| Treatment | 1 | 1703 | 1702.9 | 1.6510 | 0.2075 |
| Residuals | 34 | 35069 | 1031.4 |  |  |
|  |  |  |  |  |  |
| Estimated Marginal Means | Estimate | SE | Lower CL | Upper CL |  |
| Dun | -11.29 | 9.50 | -35.1 | 12.6 |  |
| Amphi | -2.91 | 9.50 | -26.8 | 20.9 |  |
| Tiso | 9.58 | 9.76 | -14.9 | 34.1 |  |
|  |  |  |  |  |  |
| Contrast | Estimate | SE | Df | t ratio | p value |
| Amphi - Dun | 8.38 | 12.6 | 34 | 0.665 | 0.7851 |
| Amphi - Tiso | -12.49 | 12.9 | 34 | -0.971 | 0.5998 |
| Dun - Tiso | -20.87 | 12.9 | 34 | -1.623 | 0.2501 |
|  |  |  |  |  |  |
| Percentage change in the max values of cell density (Figure 1d) | | | | | |
|  | Df | Sum Sq | Mean Sq | F value | Pr(>F) |
| Species | 2 | 1406.2 | 703.12 | 3.6469 | **0.03743** |
| Treatment | 1 | 300.6 | 300.65 | 1.5594 | 0.22082 |
| Species × treatment | 2 | 1296.0 | 647.99 | 3.3609 | **0.04733** |
| Residuals | 32 | 6169.7 | 192.80 |  |  |
|  |  |  |  |  |  |
| Estimated Marginal Means | | | | | |
| Treatment = mono |  |  |  |  |  |
| Species | Estimate | SE | Lower CL | Upper CL |  |
| Tiso | -14.8 | 8.02 | -35.027 | 5.36 |  |
| Dun | 18.1 | 8.02 | -2.118 | 38.27 |  |
| Amphi | 20.8 | 8.02 | 0.654 | 41.05 |  |
|  |  |  |  |  |  |
| Treatment = poly |  |  |  |  |  |
| Species | Estimate | SE | Lower CL | Upper CL |  |
| Tiso | 11.4 | 4.63 | -0.260 | 23.06 |  |
| Dun | 13.8 | 4.39 | 2.723 | 24.85 |  |
| Amphi | 19.3 | 4.39 | 8.192 | 30.32 |  |
|  |  |  |  |  |  |
| Contrast - Mono | Estimate | SE | Df | t ratio | p value |
| Amphi - Dun | 2.77 | 11.34 | 32 | 0.244 | 0.9676 |
| Amphi - Tiso | 35.68 | 11.34 | 32 | 3.147 | **0.0097** |
| Dun - Tiso | 32.91 | 11.34 | 32 | 2.903 | **0.0178** |
|  |  |  |  |  |  |
| Contrast - Poly | Estimate | SE | Df | t ratio | p value |
| Amphi - Dun | 5.47 | 6.21 | 32 | 0.881 | 0.6561 |
| Amphi - Tiso | 7.85 | 6.38 | 32 | 1.231 | 0.444 |
| Dun - Tiso | 2.38 | 6.38 | 32 | 0.374 | 0.9261 |
|  |  |  |  |  |  |

| **Table S2**: Linear models and post-hoc test results showing the change in max. growth rates (r_max_) and maximum value (K) of biovolume (µm^3^/µl) for each species after 9 and 17 weeks of evolution alone (mono) or in a community (poly). Initial biovolume (Biov_init) was used as a covariate. CL = 95% confidence level. Relates to Figure S1. P values < 0.05 are in bold. | | | | | | |
| --- | --- | --- | --- | --- | --- | --- |
| Species = *Amphidinium* (r_max_) – biovolume | | |  |  |  |  |
|  | | Sum Sq | df | F value | Pr(>F) |  |
| Intercept | | 1.2140e+10 | 1 | 99.7283 | **<0.0001** |  |
| Biov_init | | 1.1661e+09 | 1 | 9.3679 | **0.005727** |  |
| treatment | | 5.9700e+07 | 1 | 0.4796 | 0.495847 |  |
| Experiment | | 1.5702e+08 | 1 | 1.2614 | 0.273488 |  |
| Residuals | | 2.7385e+09 | 22 |  |  |  |
|  | |  |  |  |  |  |
| Experiment | | Estimate | SE | Lower CL | Upper CL |  |
| Week 9 | | 55959 | 3400 | 47800 | 64118 |  |
| Week 17 | | 60890 | 3437 | 52643 | 69137 |  |
|  | |  |  |  |  |  |
| Contrast | | Estimate | SE | Df | t ratio | p value |
| Week9 – Week17 | | -4931 | 4390 | 22 | -1.123 | 0.2735 |
|  | |  |  |  |  |  |
| Species = *Amphidinium* (K) – biovolume | | |  |  |  |  |
|  | | Sum Sq | df | F value | Pr(>F) |  |
| Intercept | | 1.3116e+12 | 1 | 452.4749 | **<0.0001** |  |
| Biov_init | | 1.0241e+10 | 1 | 3.5329 | 0.07347 |  |
| treatment | | 8.7004e+08 | 1 | 0.3001 | 0.58931 |  |
| Experiment | | 1.8066e+11 | 1 | 62.3240 | **<0.0001** |  |
| Residuals | | 6.3772e+10 | 21 |  |  |  |
|  | |  |  |  |  |  |
| Experiment | | Estimate | SE | Lower CL | Upper CL |  |
| Week 9 | | 767990 | 16408 | 728618 | 807363 |  |
| Week 17 | | 935249 | 16585 | 895453 | 975045 |  |
|  | |  |  |  |  |  |
| Contrast | | Estimate | SE | Df | t ratio | p value |
| Week9 – Week17 | | -167259 | 21187 | 22 | -7.895 | **<0.0001** |
|  | |  |  |  |  |  |
| Species = *Dunaliella* (r_max_) – biovolume | | |  |  |  |  |
|  | | Sum Sq | df | F value | Pr(>F) |  |
| Intercept | | 2.3852e+10 | 1 | 169.1782 | **<0.0001** |  |
| Biov_init | | 1.6560e+09 | 1 | 11.7454 | **0.002531** |  |
| treatment | | 4.3359e+08 | 1 | 3.0754 | 0.094071 |  |
| Experiment | | 4.2687e+08 | 1 | 3.0277 | 0.096488 |  |
| Treatment × Experiment | | 2.6366e+08 | 1 | 1.8701 | 0.185921 |  |
| Residuals | | 2.9607e+09 | 21 |  |  |  |
|  | |  |  |  |  |  |
| Experiment | | Estimate | SE | Lower CL | Upper CL |  |
| Week 17 | | 69192 | 3912 | 59772 | 78613 |  |
| Week 9 | | 78528 | 3912 | 69109 | 87946 |  |
|  | |  |  |  |  |  |
| Contrast | | Estimate | SE | Df | t ratio | p value |
| Week9 – Week17 | | 9335 | 5538 | 21 | 1.686 | 0.1067 |
|  | |  |  |  |  |  |
| Species = *Dunaliella* (K) – biovolume | | |  |  |  |  |
|  | | Sum Sq | df | F value | Pr(>F) |  |
| Intercept | | 3.3284e+12 | 1 | 354.8769 | **<0.0001** |  |
| Biov_init | | 3.8625e+10 | 1 | 4.1182 | 0.05469 |  |
| treatment | | 1.2582e+08 | 1 | 0.0134 | 0.90885 |  |
| Experiment | | 5.4894e+10 | 1 | 5.8528 | **0.02427** |  |
| Residuals | | 2.0634e+11 | 21 |  |  |  |
|  | |  |  |  |  |  |
| Experiment | | Estimate | SE | Lower CL | Upper CL |  |
| Week 9 | | 94588 | 29486 | 893834 | 1035342 |  |
| Week 17 | | 1056584 | 29490 | 985821 | 1127347 |  |
|  | |  |  |  |  |  |
| Contrast | | Estimate | SE | Df | t ratio | p value |
| Week9 – Week17 | | -91996 | 38027 | 22 | -2.419 | **0.0243** |
|  | |  |  |  |  |  |
| Species = *Tisochrysis* (r_max_) – biovolume | | |  |  |  |  |
|  | | Sum Sq | df | F value | Pr(>F) |  |
| Intercept | | 1.1828e+10 | 1 | 113.8353 | **<0.0001** |  |
| Biov_init | | 1.8355e+09 | 1 | 17.6651 | **0.0004377** |  |
| treatment | | 1.2408e+08 | 1 | 1.1941 | 0.2874845 |  |
| Experiment | | 2.4711e+07 | 1 | 0.2378 | 0.6310892 |  |
| Treatment × Experiment | | 5.2573e+08 | 1 | 5.0597 | **0.0359108** |  |
| Residuals | | 2.0781e+09 | 20 |  |  |  |
|  | |  |  |  |  |  |
| Experiment × treatment | | Estimate | SE | Lower CL | Upper CL |  |
| Week 9 Poly | | 60320 | 3224 | 51501 | 69138 |  |
| Week 9 Mono | | 67855 | 6102 | 51160 | 84549 |  |
| Week 17 Mono | | 72292 | 6237 | 55231 | 89353 |  |
| Week 17 Poly | | 87853 | 3400 | 78552 | 97155 |  |
|  | |  |  |  |  |  |
| Contrast | | Estimate | SE | Df | t ratio | p value |
| Week 9 Mono – Week 17 Mono | | -4437 | 9099 | 20 | -0.488 | 0.9610 |
| Week 9 Mono – Week 9 Poly | | 7535 | 6895 | 20 | 1.093 | 0.6979 |
| Week 9 Mono – Week 17 Poly | | -19999 | 6958 | 20 | -2.874 | **0.0428** |
| Week 17 Mono – Week 9 Poly | | 119782 | 7028 | 20 | 1.703 | 0.3480 |
| Week 17 Mono – Week 17 Poly | | -15562 | 7138 | 20 | -2.180 | 0.1630 |
| Week 9 Poly – Week 17 Poly | | -27534 | 4684 | 20 | -5.878 | **0.0001** |
|  | |  |  |  |  |  |
| Species = *Tisochrysis* (K) – biovolume | | |  |  |  |  |
|  | | NumDF | F value | p value |  |  |
| Intercept | | 3.3123e+12 | 1 | 413.1748 | **<0.0001** |  |
| Biov_init | | 1.4355e+10 | 1 | 1.7906 | 0.1952 |  |
| treatment | | 2.7391e+11 | 1 | 34.1671 | **<0.0001** |  |
| Experiment | | 3.4033e+11 | 1 | 42.4525 | **<0.0001** |  |
| Residuals | | 1.6835e+11 | 21 |  |  |  |
|  | |  |  |  |  |  |
| Posthoc test for treatment effect | | | | | | |
|  | | Estimate | SE | Lower CL | Upper CL |  |
| Poly | | 915811 | 20567 | 866289 | 965333 |  |
| Mono | | 1161311 | 36599 | 1073185 | 1249437 |  |
|  | |  |  |  |  |  |
| Contrast | | Estimate | SE | Df | t ratio | p value |
| Mono - Poly | | 245500 | 42000 | 21 | 5.845 | **<0.0001** |
| Posthoc test for Experiment effect | | | | | | |
|  | | Estimate | SE | Lower CL | Upper CL |  |
| Week9 | | 919773 | 27404 | 853789 | 985757 |  |
| Week17 | | 1157348 | 28183 | 1089486 | 1225210 |  |
|  | |  |  |  |  |  |
| Contrast | | Estimate | SE | Df | t ratio | p value |
| Week9 – Week17 | | -237575 | 36463 | 21 | -6.516 | **<0.0001** |
| **Table S3**: Linear models and post-hoc test results showing the change in max. growth rates (r_max_) and maximum value (K) of cell density (cells/µl) for each species after 9 and 17 weeks of evolution alone (mono) or in a community (poly). We included initial cell density as a covariate (Density_init). For the analysis of max. cell density (K) for *Tisochrysis* we used a generalised least square model with Experiment-specific variance. CL = 95% confidence level. Relates to Figure S2. P values < 0.05 are in bold. | | | | | | |
| Species = *Amphidinium* (r_max_) – cell density | | |  |  |  |  |
|  | Sum Sq | | df | F value | Pr(>F) |  |
| Intercept | 19774.3 | | 1 | 88.8262 | **<0.0001** |  |
| Density_init | 1629.8 | | 1 | 7.3211 | **0.01291** |  |
| treatment | 28.7 | | 1 | 0.1289 | 0.72297 |  |
| Experiment | 0.4 | | 1 | 0.0019 | 0.96592 |  |
| Residuals | 4897.6 | | 22 |  |  |  |
|  |  | |  |  |  |  |
| Experiment | Estimate | | SE | Lower CL | Upper CL |  |
| Week 17 | 70.4 | | 4.64 | 59.3 | 81.6 |  |
| Week 9 | 70.7 | | 4.54 | 59.8 | 81.6 |  |
|  |  | |  |  |  |  |
| Contrast | Estimate | | SE | Df | t ratio | p value |
| Week9 – Week17 | 0.256 | | 5.92 | 22 | 0.043 | 0.9659 |
|  |  | |  |  |  |  |
| Species = *Amphidinium* (K) – cell density | | |  |  |  |  |
|  | Sum Sq | | df | F value | Pr(>F) |  |
| Intercept | 2036989 | | 1 | 456.9984 | **<0.0001** |  |
| Density_init | 436 | | 1 | 0.0978 | 0.7574 |  |
| treatment | 3424 | | 1 | 0.7682 | 0.3902 |  |
| Experiment | 166482 | | 1 | 37.3502 | **<0.0001** |  |
| Resdiuals | 98061 | | 22 |  |  |  |
|  |  | |  |  |  |  |
| Experiment | Estimate | | SE | Lower CL | Upper CL |  |
| Week 9 | 869 | | 20.3 | 821 | 918 |  |
| Week 17 | 1031 | | 20.7 | 982 | 1081 |  |
|  |  | |  |  |  |  |
| Contrast | Estimate | | SE | Df | t ratio | p value |
| Week9 – Week17 | -162 | | 26.5 | 22 | -6.111 | **<0.0001** |
|  |  | |  |  |  |  |
| Species = *Dunaliella* (r_max_) – cell density | | |  |  |  |  |
|  | Sum Sq | | df | F value | Pr(>F) |  |
| Intercept | 204554 | | 1 | 111.1146 | **<0.0001** |  |
| Density_init | 7910 | | 1 | 4.2968 | 0.0501 |  |
| treatment | 249 | | 1 | 0.1353 | 0.71650 |  |
| Experiment | 5569 | | 1 | 3.0250 | 0.09596 |  |
| Resdiuals | 40500 | | 22 |  |  |  |
|  |  | |  |  |  |  |
| Experiment | Estimate | | SE | Lower CL | Upper CL |  |
| Week 17 | 182 | | 13.2 | 150 | 214 |  |
| Week 9 | 212 | | 13.1 | 180 | 243 |  |
|  |  | |  |  |  |  |
| Contrast | Estimate | | SE | Df | t ratio | p value |
| Week9 – Week17 | 29.8 | | 17.1 | 22 | 1.739 | 0.0960 |
|  |  | |  |  |  |  |
| Species = *Dunaliella* (K) – cell density | | |  |  |  |  |
|  | Sum Sq | | df | F value | Pr(>F) |  |
| Intercept | 17091821 | | 1 | 269.2690 | **<0.0001** |  |
| Density_init | 135247 | | 1 | 2.3444 | 0.13999 |  |
| treatment | 65468 | | 1 | 1.1348 | 0.29830 |  |
| Experiment | 681788 | | 1 | 11.8181 | **0.00235** |  |
| Resdiuals | 1269185 | | 22 |  |  |  |
|  |  | |  |  |  |  |
| Experiment | Estimate | | SE | Lower CL | Upper CL |  |
| Week 9 | 2103 | | 73.3 | 1927 | 2279 |  |
| Week 17 | 2433 | | 74.1 | 2255 | 2611 |  |
|  |  | |  |  |  |  |
| Contrast | Estimate | | SE | Df | t ratio | p value |
| Week9 – Week17 | -330 | | 95.9 | 22 | -3.438 | **0.0023** |
|  |  | |  |  |  |  |
| Species = *Tisochrysis* (r_max_) – cell density | | |  |  |  |  |
|  | Sum Sq | | df | F value | Pr(>F) |  |
| Intercept | 3248627 | | 1 | 193.4426 | **<0.0001** |  |
| Density_init | 319240 | | 1 | 19.0094 | **<0.0001** |  |
| treatment | 2476 | | 1 | 0.1474 | 0.7048511 |  |
| Experiment | 1740 | | 1 | 0.1036 | 0.7506994 |  |
| Resdiuals | 352669 | | 21 |  |  |  |
|  |  | |  |  |  |  |
| Experiment | Estimate | | SE | Lower CL | Upper CL |  |
| Week 9 | 988 | | 42.3 | 887 | 1090 |  |
| Week 17 | 1007 | | 41.4 | 907 | 1107 |  |
|  |  | |  |  |  |  |
| Contrast | Estimate | | SE | Df | t ratio | p value |
| Week9 – Week17 | -18.4 | | 57.3 | 21 | -0.322 | 0.7507 |
|  |  | |  |  |  |  |
| Species = *Tisochrysis* (K) – cell density | | |  |  |  |  |
|  | NumDF | | F value | P value |  |  |
| Intercept | 1 | | 4383.954 | **<0.0001** |  |  |
| Density_init | 1 | | 6.581 | **0.0185** |  |  |
| Experiment | 1 | | 1.551 | 0.2274 |  |  |
| Treatment | 1 | | 39.257 | **<0.0001** |  |  |
| Experiment × treatment | 1 | | 15.131 | **0.0009** |  |  |
|  |  | |  |  |  |  |
| Experiment = Week 9 | Estimate | | SE | Lower CL | Upper CL |  |
| Mono | 11408 | | 428 | 10353 | 12462 |  |
| Poly | 15649 | | 434 | 14130 | 17168 |  |
|  |  | |  |  |  |  |
| Experiment = Week 17 | Estimate | | SE | Lower CL | Upper CL |  |
| Mono | 12089 | | 462 | 10966 | 13229 |  |
| Poly | 13148 | | 378 | 11611 | 14685 |  |
|  |  | |  | cbri |  |  |
|  |  | |  |  |  |  |
| Contrast | Estimate | | SE | Df | t ratio | p value |
| Week 9 – Mono - Poly | 4247 | | 590 | 12.6 | 7.192 | **<0.0001** |
| Week 17– Mono - Poly | 1050 | | 584 | 13.7 | 1.798 | 0.0942 |

| **Table S4:** Linear model and post-hoc test results on changes in cell size between the ancestors and day 0 of each common garden experiment. For this analysis the factor “Condition” combines Experiment (Week 9, Week 17) and competition treatment (monoculture, polyculture). CL = 95% confidence level. P values < 0.05 are in bold. Related to Figure S5. | | | | | |
| --- | --- | --- | --- | --- | --- |
| Cell Size | | | | | |
|  | Df | Sum Sq | Mean Sq | F value | Pr(>F) |
| Species | 2 | 91.113 | 45.557 | 27936.175 | **<0.0001** |
| Condition | 4 | 0.999 | 0.25 | 152.107 | **<0.0001** |
| Species × Condition | 8 | 0.137 | 0.017 | 10.422 | **<0.0001** |
| Residuals | 425 | 0.703 | 0.002 |  | **<0.0001** |
|  |  |  |  |  |  |
| Posthoc test for Experiment × species |  |  |  |  |  |
| Species = *Amphidinium* |  |  |  |  |  |
| Condition | Estimate | SE | Lower CL | Upper CL |  |
| Ancestor | 2.975 | 0.003668 | 2.968 | 2.982 |  |
| Week 9_Mono | 2.897 | 0.023394 | 2.851 | 2.943 |  |
| Week 9_Poly  Week 17_Mono | 2.932  2.908 | 0.12813  0.023394 | 2.907  2.862 | 2.957  2.954 |  |
| Week 17_Poly | 2.882 | 0.012813 | 2.857 | 2.907 |  |
| Contrast | Estimate | SE | Df | t ratio | p value |
| Ancestor – Week 9_Mono | 0.0779 | 0.0237 | 428 | 3.29 | **0.0095** |
| Ancestor – Week 9_Poly | 0.0426 | 0.0133 | 428 | 3.194 | **0.013** |
| Ancestor – Week 17_Mono | 0.0663 | 0.0237 | 428 | 2.799 | **0.0424** |
| Ancestor – Week 17_Poly | 0.093 | 0.0133 | 428 | 6.977 | **<0.0001** |
| Week 9_Mono - Week 9_Poly | -0.0353 | 0.0267 | 428 | -1.325 | 0.6758 |
| Week 9_Mono - Week 17_Mono | -0.0116 | 0.0331 | 428 | -0.351 | 0.9967 |
| Week 9_Mono - Week 17_Poly | 0.0151 | 0.0267 | 428 | 0.565 | 0.98 |
| Week 9_Poly - Week 17_Mono | 0.0237 | 0.0267 | 428 | 0.889 | 0.9009 |
| Week 9_Poly - Week 17_Poly | 0.0504 | 0.0181 | 428 | 2.782 | 0.0445 |
| Week 17_Mono - Week 17_Poly | 0.0267 | 0.0267 | 428 | 1.001 | 0.8549 |
|  |  |  |  |  |  |
| Species = *Dunaliella* |  |  |  |  |  |
| Condition | Estimate | SE | Lower CL | Upper CL |  |
| Ancestor | 2.794 | 0.003668 | 2.787 | 2.801 |  |
| Week 9_Mono | 2.61 | 0.023394 | 2.564 | 2.656 |  |
| Week 9_Poly  Week 17_Mono | 2.655  2.567 | 0.012813  0.023394 | 2.63  2.521 | 2.68  2.613 |  |
| Week 17_Poly | 2.592 | 0.012813 | 2.567 | 2.617 |  |
|  |  |  |  |  |  |
| Contrast | Estimate | SE | Df | t ratio | p value |
| Ancestor – Week 9_Mono | 0.1837 | 0.0237 | 428 | 7.759 | **<0.0001** |
| Ancestor – Week 9_Poly | 0.1386 | 0.0133 | 428 | 10.396 | **<0.0001** |
| Ancestor – Week 17_Mono | 0.2272 | 0.0237 | 428 | 9.595 | **<0.0001** |
| Ancestor – Week 17_Poly | 0.2014 | 0.0133 | 428 | 15.113 | **<0.0001** |
| Week 9_Mono - Week 9_Poly | -0.0452 | 0.0267 | 428 | -1.694 | 0.439 |
| Week 9_Mono - Week 17_Mono | 0.0435 | 0.0331 | 428 | 1.314 | 0.6825 |
| Week 9_Mono - Week 17_Poly | 0.0177 | 0.0267 | 428 | 0.664 | 0.964 |
| Week 9_Poly - Week 17_Mono | 0.0887 | 0.0267 | 428 | 3.324 | **0.0085** |
| Week 9_Poly - Week 17_Poly | 0.0629 | 0.0181 | 428 | 3.47 | **0.0052** |
| Week 17_Mono - Week 17_Poly | -0.0258 | 0.0267 | 428 | -0.966 | 0.8701 |
|  |  |  |  |  |  |
| Species = *Tisochrysis* |  |  |  |  |  |
| Condition | Estimate | SE | Lower CL | Upper CL |  |
| Ancestor | 1.932 | 0.003668 | 1.925 | 1.939 |  |
| Week 9_Mono | 1.828 | 0.023394 | 1.782 | 1.874 |  |
| Week 9_Poly  Week 17_Mono | 1.851  1.807 | 0.012813  0.023394 | 1.826  1.761 | 1.876  1.853 |  |
| Week 17_Poly | 1.773 | 0.013507 | 1.747 | 1.8 |  |
|  |  |  |  |  |  |
| Contrast | Estimate | SE | Df | t ratio | p value |
| Ancestor – Week 9_Mono | 0.1042 | 0.0237 | 428 | 4.401 | **0.0001** |
| Ancestor – Week 9_Poly | 0.081 | 0.0133 | 428 | 6.078 | **<0.0001** |
| Ancestor – Week 17_Mono | 0.1245 | 0.0237 | 428 | 5.256 | **<0.0001** |
| Ancestor – Week 17_Poly | 0.1585 | 0.014 | 428 | 11.322 | **<0.0001** |
| Week 9_Mono - Week 9_Poly | -0.0232 | 0.0267 | 428 | -0.87 | 0.9079 |
| Week 9_Mono - Week 17_Mono | 0.0202 | 0.0331 | 428 | 0.612 | 0.9732 |
| Week 9_Mono - Week 17_Poly | 0.0543 | 0.027 | 428 | 2.009 | 0.2635 |
| Week 9_Poly - Week 17_Mono | 0.0434 | 0.0267 | 428 | 1.629 | 0.4797 |
| Week 9_Poly - Week 17_Poly | 0.0775 | 0.0186 | 428 | 4.161 | **0.0004** |
| Week 17_Mono - Week 17_Poly | 0.034 | 0.027 | 428 | 1.259 | 0.7163 |
|  |  |  |  |  |  |

| **Table S5**: Linear models and post-hoc test results of the changes in cell size throughout each common garden experiment (week 9, week 17), competition treatment (mono, poly) and experiment day (0 to 25). Relates to Figure S4. P values < 0.05 are in bold. | | | | | |
| --- | --- | --- | --- | --- | --- |
| Species = *Amphidinium* |  |  |  |  |  |
|  | Df | Sum Sq | Mean Sq | F value | Pr(>F) |
| Experiment | 1 | 87824 | 87824 | 25.102 | **<0.0001** |
| Treatment | 1 | 277 | 277 | 0.0792 | 0.77862 |
| Exp_day | 12 | 202549 | 16879 | 4.8244 | **<0.0001** |
| Experiment × treatment | 1 | 6333 | 6333 | 1.8101 | 0.17961 |
| Experiment × exp_day | 11 | 326841 | 29713 | 8.4926 | **<0.0001** |
| Treatment × exp_day | 12 | 25588 | 2132 | 0.6095 | 0.83375 |
| Experiment × treatment × exp_day | 11 | 67980 | 6180 | 1.7664 | 0.05972 |
| Residuals | 274 | 958636 | 3499 |  |  |
|  |  |  |  |  |  |
| Species = *Dunaliella* |  |  |  |  |  |
|  | Df | Sum Sq | Mean Sq | F value | Pr(>F) |
| Experiment | 1 | 11613 | 11612.6 | 19.0158 | **<0.0001** |
| Treatment | 1 | 2699 | 2698.5 | 4.4188 | **0.03516** |
| Exp_day | 12 | 173588 | 14465.7 | 24.015 | **<0.0001** |
| Experiment × exp_day | 11 | 91494 | 8317.6 | 13.8084 | **<0.0001** |
| Treatment × exp_day | 12 | 12356 | 1029.7 | 1.7095 | 0.06418 |
| Residuals | 287 | 172877 | 602.4 |  |  |
|  |  |  |  |  |  |
| Species = *Tisochrysis* |  |  |  |  |  |
|  | Df | Sum Sq | Mean Sq | F value | Pr(>F) |
| Experiment | 1 | 15655.8 | 15655.8 | 488.3005 | **<0.0001** |
| Treatment | 1 | 239.9 | 239.9 | 7.481 | **0.006661** |
| Exp_day | 12 | 9172 | 764.3 | 23.8394 | **<0.0001** |
| Experiment × treatment | 1 | 30.2 | 30.2 | 0.9431 | 0.332383 |
| Experiment × exp_day | 11 | 10364.8 | 942.3 | 29.3888 | **<0.0001** |
| Treatment × exp_day | 12 | 231.6 | 19.3 | 0.6018 | 0.840007 |
| Experiment × treatment × exp_day | 11 | 463.1 | 42.1 | 1.3131 | 0.216839 |
| Residuals | 262 | 8400.2 | 32.1 |  |  |

| **Table S6**: Linear models and post-hoc test results showing the relationship between the intrinsic rate of increase (r) and the intraspecific competition coefficient 𝛼_ii_ for each common garden experiment (week 9, week 17) and competition treatment (monoculture, polyculture). Both r and 𝛼_ii_ are calculated from a growth model fitted on biovolume data. CL = 95% confidence level. Relates to Figure 3a. P values < 0.05 are in bold. | | | | | | |
| --- | --- | --- | --- | --- | --- | --- |
|  | | Df | Sum Sq | Mean Sq | F value | Pr(>F) |
| r | | 1 | 8.1790e-14 | 8.1790e-14 | 7.0151 | **0.009147** |
| Experiment | | 1 | 5.7324e-13 | 5.7324e-13 | 49.1686 | **<0.0001** |
| Treatment | | 1 | 1.0369e-13 | 1.0369e-13 | 8.8938 | **0.003779** |
| species | | 2 | 9.9598e-13 | 4.9799e-13 | 42.7143 | **<0.0001** |
| r × species | | 2 | 4.5700e-14 | 2.2850e-14 | 1.96 | 0.147487 |
| Experiment × treatment | | 1 | 1.7200e-14 | 1.7200e-14 | 1.4749 | 0.228102 |
| Experiment × species | | 2 | 1.0862e-13 | 5.4310e-14 | 4.6582 | **0.012164** |
| Treatment × species | | 2 | 4.5510e-14 | 2.2750e-14 | 1.9517 | 0.148648 |
| Residuals | | 81 | 9.4435e-13 | 1.1660e-14 |  |  |
|  | |  |  |  |  |  |
| Posthoc test for Experiment × species | | |  |  |  |  |
| Species = *Amphidinium* | | |  |  |  |  |
| Experiment | Estimate | | SE | Lower CL | Upper CL |  |
| Week 9 | 1.40e-06 | | 3.17e-08 | 1.33e-06 | 1.46e-06 |  |
| Week 17 | 1.20e-06 | | 2.79e-08 | 1.15e-06 | 1.26e-06 |  |
|  |  | |  |  |  |  |
| Species = *Dunaliella* |  | |  |  |  |  |
| Experiment | Estimate | | SE | Lower CL | Upper CL |  |
| Week 9 | 1.11e-06 | | 2.98e-08 | 1.05e-06 | 1.17e-06 |  |
| Week 17 | 1.04e-06 | | 2.76e-08 | 9.87e-07 | 1.10e-06 |  |
|  |  | |  |  |  |  |
| Species = *Tisochrysis* |  | |  |  |  |  |
| Experiment | Estimate | | SE | Lower CL | Upper CL |  |
| Week 9 | 1.18e-06 | | 2.85e-08 | 1.12e-06 | 1.23e-06 |  |
| Week 17 | 9.54e-07 | | 2.83e-08 | 8.97e-07 | 1.01e-06 |  |
|  |  | |  |  |  |  |
| Species =*Amphidinium* | | | | | | |
| Contrast | | Estimate | SE | Df | t ratio | p value |
| Week9 – Week17 | | 1.97e-07 | 4.33e-08 | 81 | 4.555 | **<0.0001** |
|  | |  |  |  |  |  |
| Species =*Dunaliella* | |  |  |  |  |  |
| Contrast | | Estimate | SE | Df | t ratio | p value |
| Week9 – Week17 | | 6.86e-08 | 4.06e-08 | 81 | 1.691 | 0.0948 |
|  | |  |  |  |  |  |
| Species =*Tisochrysis* | |  |  |  |  |  |
| Contrast | | Estimate | SE | Df | t ratio | p value |
| Week9 – Week17 | | 2.24e-07 | 3.95e-08 | 81 | 5.664 | **<0.0001** |
|  | |  |  |  |  |  |
| Posthoc test for Competition Treatment | | |  |  |  |  |
| Treatment | Estimate | | SE | Lower CL | Upper CL |  |
| Mono | 1.12e-06 | | 1.93e-08 | 1.08e-06 | 1.16e-06 |  |
| Poly | 1.18e-06 | | 1.43e-08 | 1.15e-06 | 1.20e-06 |  |
|  |  | |  |  |  |  |
| Competition Treatment | |  |  |  |  |  |
| Contrast | | Estimate | SE | Df | t ratio | p value |
| Mono - Poly | | -5.78e-08 | 2.46e-08 | 81 | -2.349 | **0.0213** |

| **Table S7**: Linear mixed effect models and post-hoc testing for changes in *per capita* net energy production (J/day) with population biovolume (biov; μm^3^/μl) (both log_10_-transformed) between competition treatments (monoculture, polyculture), experiments (week 9, week 17) and species (*Amphidinium*, *Dunaliella*, *Tisochrysis*). CL = 95% confidence level. P values < 0.05 are in bold. Related to Figure 3b and Figure S6. | | | | | | | | | | | | | | | | |
| --- | --- | --- | --- | --- | --- | --- | --- | --- | --- | --- | --- | --- | --- | --- | --- | --- |
|  | | | | Sum Sq | | | Mean Sq | | DF | | | DenDF | F value | | | Pr(>F) |
| log_10_(biov) | | | | 10.6951 | | | 10.6951 | | 1 | | | 644.57 | 414.1516 | | | **<0.0001** |
| Experiment | | | | 2.5178 | | | 2.5178 | | 1 | | | 646.64 | 97.4962 | | | **<0.0001** |
| treatment | | | | 0.01287 | | | 0.0128 | | 1 | | | 646.64 | 0.4964 | | | 0.481334 |
| species | | | | 0.0909 | | | 0.0455 | | 2 | | | 646.57 | 1.7606 | | | 0.172772 |
| log_10_ (biov) × Experiment | | | | 2.7850 | | | 2.7850 | | 1 | | | 644.57 | 107.8456 | | | **<0.0001** |
| log_10_ (biov) × treatment | | | | 0.0153 | | | 0.0153 | | 1 | | | 644.57 | 0.5917 | | | 0.442051 |
| Experiment × treatment | | | | 0.0866 | | | 0.0866 | | 1 | | | 646.64 | 3.3535 | | | 0.067523 |
| log_10_ (biov) × species | | | | 0.3232 | | | 0.1616 | | 2 | | | 644.49 | 6.2581 | | | **0.002033** |
| Experiment × species | | | | 0.5247 | | | 0.2624 | | 2 | | | 646.57 | 10.1598 | | | **<0.0001** |
| Treatment × species | | | | 0.0305 | | | 0.0153 | | 2 | | | 646.57 | 0.5910 | | | 0.554091 |
| log_10_ (biov) × Experiment × treatment | | | | 0.0750 | | | 0.0750 | | 1 | | | 644.50 | 2.9030 | | | *0.088896(*)* |
| log_10_ (biov) × Experiment × species | | | | 0.5764 | | | 0.2882 | | 2 | | | 644.50 | 11.1606 | | | **<0.001** |
| log_10_ (biov) × treatment × species | | | | 0.0290 | | | 0.0145 | | 2 | | | 644.49 | 0.5621 | | | 0.570292 |
| Experiment × treatment × species | | | | 0.0825 | | | 0.0413 | | 2 | | | 646.57 | 1.5976 | | | 0.203174 |
| log_10_(biov) × Experiment × treatment × species | | | | 0.0803 | | | 0.0402 | | 2 | | | 644.50 | 1.5553 | | | 0.211911 |
|  | | | | | | | | | | | | | | | |  |
| Posthoc test for biovolume × Experiment × Species | | | | | | | | | | | | | | | | |
| Species | | | Experiment | | | | | | | | Slope estimates and CL | | | | | |
| *Amphidinium* | | | Week 9 | | | | | | | | -0.979 (-1.124, -0.8344) | | | | | |
|  | | | Week 17 | | | | | | | | -0.0508 (-0.192, -0.0907) | | | | | |
|  | | |  | | | | | | | |  | | | | | |
| *Dunaliella* | | | Week 9 | | | | | | | | -0.7601 (-0.918, -0.6027) | | | | | |
|  | | | Week 17 | | | | | | | | -0.5205 (-0.677, -0.3641) | | | | | |
|  | | |  | | | | | | | |  | | | | | |
| *Tisochrysis* | | | Week 9 | | | | | | | | -1.1943 (-1.350, -1.0389) | | | | | |
|  | | | Week 17 | | | | | | | | -0.3801 (-0.542, -0.2183) | | | | | |
|  | | |  | | | | | | | |  | | | | | |
| Species = *Amphidinium* | | | | | | | | | | | | | | | | |
| Contrast | | Estimate | | | SE | | | Df | | t ratio | | | | p value | |  |
| Week9 – Week17 | | -0.929 | | | 0.103 | | | 644 | | -9.000 | | | | **<0.0001** | |  |
|  | |  | | |  | | |  | |  | | | |  | |  |
| Species = *Dunaliella* | |  | | |  | | |  | |  | | | |  | | |
| Contrast | | Estimate | | | SE | | | Df | | t ratio | | | | p value | |  |
| Week9 – Week17 | | -0.270 | | | 0.113 | | | 644 | | -2.121 | | | | **0.0343** | |  |
|  | |  | | |  | | |  | |  | | | |  | |  |
|  | |  | | |  | | |  | |  | | | |  | | |
| Species = *Tisochrysis* | |  | | |  | | |  | |  | | | |  | | |
| Contrast | | Estimate | | | SE | | | Df | | t ratio | | | | p value | |  |
| Week9 – Week17 | | -0.814 | | | 0.114 | | | 650 | | -7.127 | | | | **<0.0001** | |  |
|  | |  | | |  | | |  | |  | | | |  | |  |
| Posthoc test for biovolume × Experiment × Treatment (p = 0.09) *(*)* | | | | | | | | | | | | | | | | |
| Experiment | | | Competition treatment | | | | | | | | Slope estimates and CL | | | | | |
| Week 9 | | | Mono | | | | | | | | -1.008 (-1.166, -0.8497) | | | | | |
|  | | | Poly | | | | | | | | -0.948 (-1.027, -0.8699) | | | | | |
|  | | |  | | | | | | | |  | | | | | |
|  | | |  | | | | | | | |  | | | | | |
| Week 17 | | | Mono | | | | | | | | -0.238 (-0.393, -0.0835) | | | | | |
|  | | | Poly | | | | | | | | -0.396 (-0.482, -0.3099) | | | | | |
|  |  | |  | | |  | | | | |  | | | |  | |
| Experiment = Week 9 | | | | | | | | | | | | | | | | |
| Contrast | Estimate | | SE | | | Df | | | | | t ratio | | | | p value | |
| Mono – Poly | -0.0595 | | 0.0899 | | | 641 | | | | | -0.662 | | | | 0.5083 | |
|  |  | |  | | |  | | | | |  | | | |  | |
| Experiment = Week 17 | | | | | | | | | | | | | | | | |
| Contrast | Estimate | | SE | | | Df | | | | | t ratio | | | | p value | |
| Mono – Poly | 0.1574 | | 0.0902 | | | 651 | | | | | 1.744 | | | | 0.0816 | |

| **Table S8**: Linear model and post-hoc test results on changes in *per capita* photosynthesis and respiration rates (J/min, log_10_-transformed) between species, condition (experiment and competition treatment combined) and growth phase (day 4 in the middle of exponential phase vs day 25 in stationary phase). Related to Figure 4. CL = 95% confidence level. P values < 0.05 are in bold. | | | | | |
| --- | --- | --- | --- | --- | --- |
| Photosynthesis | | | | | |
|  | Df | Sum Sq | Mean Sq | F value | Pr(>F) |
| Species | 2 | 21.7368 | 10.8684 | 441.456 | **<0.001** |
| Condition (experiment and treatment combined) | 3 | 0.8226 | 0.2742 | 11.137 | **<0.001** |
| Phase (Day 4 vs Day 25) | 1 | 4.8380 | 4.8380 | 196.5127 | **<0.001** |
| Species × condition | 6 | 0.0688 | 0.0115 | 0.4658 | 0.832596 |
| Species × phase | 2 | 0.2454 | 0.1227 | 4.49838 | **0.008214** |
| Condition × phase | 3 | 0.3178 | 0.1059 | 4.3023 | **0.006268** |
| Species × condition × phase | 6 | 0.3126 | 0.0521 | 2.1161 | **0.055633** |
| Residuals | 130 | 3.2005 | 0.0246 |  |  |
|  |  |  |  |  |  |
| Posthoc test for species × condition × phase | | | | | |
| Species = *Amphidinium*, Phase = Exponential | | | | | |
| Condition | Estimate | SE | Lower CL | Upper CL |  |
| Week 9_Mono | -8.31 | 0.0906 | -8.48 | -8.13 |  |
| Week 9_Poly | -8.25 | 0.0496 | -8.35 | -8.15 |  |
| Week 17_Mono | -8.29 | 0.0906 | -8.46 | -8.11 |  |
| Week 17_Poly | -8.15 | 0.0496 | -8.25 | -8.06 |  |
|  |  |  |  |  |  |
| Species = *Amphidinium*, Phase = Stationary | | | | |  |
| Condition | Estimate | SE | Lower CL | Upper CL |  |
| Week 9_Mono | -8.59 | 0.0906 | -8.77 | -8.41 |  |
| Week 9_Poly | -8.63 | 0.0496 | -8.73 | -8.54 |  |
| Week 17_Mono | -8.41 | 0.0906 | -8.59 | -8.23 |  |
| Week 17_Poly | -8.4 | 0.0496 | -8.49 | -8.3 |  |
|  |  |  |  |  |  |
| Species = *Dunaliella*, Phase = Exponential | | | | | |
| Condition | Estimate | SE | Lower CL | Upper CL |  |
| Week 9_Mono | -8.59 | 0.0906 | -8.77 | -8.41 |  |
| Week 9_Poly | -8.59 | 0.0496 | -8.69 | -8.49 |  |
| Week 17_Mono | -8.5 | 0.0906 | -8.68 | -8.32 |  |
| Week 17_Poly | -8.4 | 0.0496 | -8.5 | -8.3 |  |
|  |  |  |  |  |  |
| Species = *Dunaliella*, Phase = Stationary | | | | | |
| Condition | Estimate | SE | Lower CL | Upper CL |  |
| Week 9_Mono | -8.9 | 0.0906 | -9.08 | -8.72 |  |
| Week 9_Poly | -8.92 | 0.0496 | -9.02 | -8.82 |  |
| Week 17_Mono | -8.76 | 0.0906 | -8.94 | -8.58 |  |
| Week 17_Poly | -8.71 | 0.0496 | -8.81 | -8.61 |  |
|  |  |  |  |  |  |
| Species = *Tisochrysis*, Phase = Exponential | | | | | |
| Condition | Estimate | SE | Lower CL | Upper CL |  |
| Week 9_Mono | -8.9 | 0.0906 | -9.08 | -8.72 |  |
| Week 9_Poly | -9.03 | 0.0496 | -9.13 | -8.93 |  |
| Week 17_Mono | -9.11 | 0.0906 | -9.29 | -8.93 |  |
| Week 17_Poly | -9.08 | 0.0523 | -9.18 | -8.97 |  |
|  |  |  |  |  |  |
| Species = *Tisochrysis*, Phase = Stationary | | | | | |
| Condition | Estimate | SE | Lower CL | Upper CL |  |
| Week 9_Mono | -9.81 | 0.0906 | -9.99 | -9.63 |  |
| Week 9_Poly | -9.58 | 0.0496 | -9.68 | -9.49 |  |
| Week 17_Mono | -9.4 | 0.0906 | -9.58 | -9.22 |  |
| Week 17_Poly | -9.36 | 0.0523 | -9.47 | -9.26 |  |
|  |  |  |  |  |  |
| Phase = exponential, species = *Amphidinium* | | | | | |
| Contrast | Estimate | SE | Df | t ratio | p value |
| Week 9_Mono - Week 9_Poly | -0.05312 | 0.1033 | 130 | -0.514 | 0.9556 |
| Week 9_Mono - Week 17_Mono | -0.0206 | 0.1281 | 130 | -0.161 | 0.9985 |
| Week 9_Mono - Week 17_Poly | -0.15113 | 0.1033 | 130 | -1.463 | 0.4627 |
| Week 9_Poly - Week 17_Mono | 0.03252 | 0.1033 | 130 | 0.315 | 0.9891 |
| Week 9_Poly - Week 17_Poly | -0.09801 | 0.0702 | 130 | -1.397 | 0.5037 |
| Week 17_Mono - Week 17_Poly | -0.13053 | 0.1033 | 130 | -1.264 | 0.5876 |
|  |  |  |  |  |  |
| Phase = stationary, species = *Amphidinium* | | | | | |
| Contrast | Estimate | SE | Df | t ratio | p value |
| Week 9_Mono - Week 9_Poly | 0.04381 | 0.1033 | 130 | 0.424 | 0.9743 |
| Week 9_Mono - Week 17_Mono | -0.1826 | 0.1281 | 130 | -1.425 | 0.4859 |
| Week 9_Mono - Week 17_Poly | -0.19459 | 0.1033 | 130 | -1.884 | 0.24 |
| Week 9_Poly - Week 17_Mono | -0.22642 | 0.1033 | 130 | -2.192 | 0.1309 |
| Week 9_Poly - Week 17_Poly | -0.23841 | 0.0702 | 130 | -3.398 | **0.0049** |
| Week 17_Mono - Week 17_Poly | -0.01199 | 0.1033 | 130 | -0.116 | 0.9994 |
|  |  |  |  |  |  |
| Phase = exponential, species = *Dunaliella* | | | | | |
| Contrast | Estimate | SE | Df | t ratio | p value |
| Week 9_Mono - Week 9_Poly | 0.00455 | 0.1033 | 130 | 0.044 | 1 |
| Week 9_Mono - Week 17_Mono | -0.0875 | 0.1281 | 130 | -0.683 | 0.9034 |
| Week 9_Mono - Week 17_Poly | -0.18613 | 0.1033 | 130 | -1.802 | 0.2771 |
| Week 9_Poly - Week 17_Mono | -0.09205 | 0.1033 | 130 | -0.891 | 0.8094 |
| Week 9_Poly - Week 17_Poly | -0.19068 | 0.0702 | 130 | -2.717 | **0.0371** |
| Week 17_Mono - Week 17_Poly | -0.09863 | 0.1033 | 130 | -0.955 | 0.7752 |
|  |  |  |  |  |  |
| Phase = stationary, species = *Dunaliella* | | | | | |
| Contrast | Estimate | SE | Df | t ratio | p value |
| Week 9_Mono - Week 9_Poly | 0.02427 | 0.1033 | 130 | 0.235 | 0.9954 |
| Week 9_Mono - Week 17_Mono | -0.1377 | 0.1281 | 130 | -1.075 | 0.7055 |
| Week 9_Mono - Week 17_Poly | -0.18782 | 0.1033 | 130 | -1.818 | 0.2694 |
| Week 9_Poly - Week 17_Mono | -0.16197 | 0.1033 | 130 | -1.568 | 0.4004 |
| Week 9_Poly - Week 17_Poly | -0.21208 | 0.0702 | 130 | -3.022 | **0.0158** |
| Week 17_Mono - Week 17_Poly | -0.05011 | 0.1033 | 130 | -0.485 | 0.9623 |
|  |  |  |  |  |  |
| Phase = exponential, species = *Tisochrysis* | | | | | |
| Contrast | Estimate | SE | Df | t ratio | p value |
| Week 9_Mono - Week 9_Poly | 0.13353 | 0.1033 | 130 | 1.293 | 0.5691 |
| Week 9_Mono - Week 17_Mono | 0.21231 | 0.1281 | 130 | 1.657 | 0.3506 |
| Week 9_Mono - Week 17_Poly | 0.18131 | 0.1046 | 130 | 1.733 | 0.3108 |
| Week 9_Poly - Week 17_Mono | 0.07878 | 0.1033 | 130 | 0.763 | 0.871 |
| Week 9_Poly - Week 17_Poly | 0.04777 | 0.0721 | 130 | 0.663 | 0.9109 |
| Week 17_Mono - Week 17_Poly | -0.03101 | 0.1046 | 130 | -0.296 | 0.9909 |
|  |  |  |  |  |  |
| Phase = stationary, species = *Tisochrysis* | | | | | |
| Contrast | Estimate | SE | Df | t ratio | p value |
| Week 9_Mono - Week 9_Poly | -0.22491 | 0.1033 | 130 | -2.178 | 0.135 |
| Week 9_Mono - Week 17_Mono | -0.40739 | 0.1281 | 130 | -3.18 | **0.0098** |
| Week 9_Mono - Week 17_Poly | -0.44544 | 0.1046 | 130 | -4.258 | **0.0002** |
| Week 9_Poly - Week 17_Mono | -0.18248 | 0.1033 | 130 | -1.767 | 0.2941 |
| Week 9_Poly - Week 17_Poly | -0.22053 | 0.0721 | 130 | -3.059 | **0.0142** |
| Week 17_Mono - Week 17_Poly | -0.03804 | 0.1046 | 130 | -0.364 | 0.9835 |
|  |  |  |  |  |  |
| Respiration | | | | | |
|  | Df | Sum Sq | Mean Sq | F value | Pr(>F) |
| Species | 2 | 35.964 | 17.819 | 176.1180 | **<0.0001** |
| Condition (experiment and treatment combined) | 3 | 1.418 | 0.4727 | 4.6295 | **0.004117** |
| Phase (Day 4 vs Day 25) | 1 | 1.673 | 1.6727 | 16.3830 | **<0.0001** |
| Condition × phase | 3 | 1.127 | 0.3758 | 3.6802 | **0.013822** |
| Residuals | 132 | 13.477 | 0.1021 |  |  |
|  |  |  |  |  |  |
| Posthoc test for condition × phase | | | | | |
| Phase = Exponential |  |  |  |  |  |
| Condition | Estimate | SE | Lower CL | Upper CL |  |
| Week 9_Mono | -9.55 | 0.1065 | -9.76 | -9.34 |  |
| Week 9_Poly | -9.52 | 0.0583 | -9.63 | -9.4 |  |
| Week 17_Mono | -9.73 | 0.1209 | -9.97 | -9.49 |  |
| Week 17_Poly | -9.98 | 0.0743 | -10.13 | -9.84 |  |
|  |  |  |  |  |  |
| Phase = Stationary |  |  |  |  |  |
| Condition | Estimate | SE | Lower CL | Upper CL |  |
| Week 9_Mono | -9.54 | 0.1065 | -9.75 | -9.33 |  |
| Week 9_Poly | -9.43 | 0.0583 | -9.54 | -9.31 |  |
| Week 17_Mono | -9.48 | 0.1065 | -9.69 | -9.26 |  |
| Week 17_Poly | -9.52 | 0.0594 | -9.64 | -9.40 |  |
|  |  |  |  |  |  |
| Phase = Exponential |  |  |  |  |  |
| Contrast | Estimate | SE | Df | t ratio | p value |
| Week 9_Mono - Week 9_Poly | -0.0344 | 0.1214 | 132 | -0.283 | 0.992 |
| Week 9_Mono - Week 17_Mono | 0.1835 | 0.1611 | 132 | 1.139 | 0.6662 |
| Week 9_Mono - Week 17_Poly | 0.4333 | 0.1299 | 132 | 3.336 | **0.006** |
| Week 9_Poly - Week 17_Mono | 0.2179 | 0.1342 | 132 | 1.623 | 0.3692 |
| Week 9_Poly - Week 17_Poly | 0.4677 | 0.0945 | 132 | 4.95 | **<0.0001** |
| Week 17_Mono - Week 17_Poly | 0.2498 | 0.1419 | 132 | 1.76 | 0.2975 |
|  |  |  |  |  |  |
| Phase = Stationary |  |  |  |  |  |
| Contrast | Estimate | SE | Df | t ratio | p value |
| Week 9_Mono - Week 9_Poly | -0.1158 | 0.1214 | 132 | -0.953 | 0.776 |
| Week 9_Mono - Week 17_Mono | -0.0669 | 0.1506 | 132 | -0.444 | 0.9707 |
| Week 9_Mono - Week 17_Poly | -0.0212 | 0.1219 | 132 | -0.174 | 0.9981 |
| Week 9_Poly - Week 17_Mono | 0.0489 | 0.1214 | 132 | 0.403 | 0.9778 |
| Week 9_Poly - Week 17_Poly | 0.0946 | 0.0832 | 132 | 1.137 | 0.6674 |
| Week 17_Mono - Week 17_Poly | 0.0457 | 0.1219 | 132 | 0.375 | 0.982 |
|  |  |  |  |  |  |
| Posthoc test for main species effect |  |  |  |  |  |
| Species | Estimate | SE | Lower CL | Upper CL |  |
| *Amphidinium* | -9.01 | 0.0515 | -9.11 | -8.91 |  |
| *Dunaliella* | -9.54 | 0.0484 | -9.63 | -9.44 |  |
| *Tisochrysis* | -10.23 | 0.0492 | -10.33 | -10.14 |  |
|  |  |  |  |  |  |
| Contrast | Estimate | SE | Df | t ratio | p value |
| *Amphidinium* - *Dunaliella* | 0.526 | 0.0655 | 132 | 7.905 | **<0.0001** |
| *Amphidinium* - *Tisochrysis* | 1.223 | 0.0673 | 132 | 18.182 | **<0.0001** |
| *Dunaliella* - *Tisochrysis* | 0.697 | 0.0649 | 132 | 10.740 | **<0.0001** |
|  |  |  |  |  |  |

| **Table S9**: Linear Mixed-Effects Model and post-hoc tests for changes in population growth (cells per day; log_10_-transformed) as populations approach carrying capacity (day 8 onwards) considering the effect of experiment day (8 to 25), experiment (Week 9, Week 17), competition treatment (monoculture, polyculture) and species (*Amphidinium, Dunaliella, Tisochrysis*). We included the unique sample ID as random effect. CL = 95% confidence level. Relates to Figure S7. P values < 0.05 are in bold. | | | | | | |
| --- | --- | --- | --- | --- | --- | --- |
| Species = *Amphidinium* |  |  |  |  |  |  |
|  | Sum Sq | Mean Sq | NumDF | DenDF | F value | Pr(>F) |
| Exp_day  Experiment | 62.887  0.393 | 62.887  0.393 | 1  1 | 410.47  241.28 | 1063.673  6.644 | **<0.0001**  **0.010544** |
| Treatment | 0.372 | 0.372 | 1 | 241.28 | 6.2845 | **0.012838** |
| Species | 3.846 | 1.923 | 2 | 241.28 | 32.5302 | **<0.0001** |
| Exp_day × Experiment | 2.426 | 2.426 | 1 | 410.47 | 41.0396 | **<0.0001** |
| Exp_day × treatment | 1.866 | 1.866 | 1 | 410.47 | 31.5665 | **<0.0001** |
| Experiment × treatment | 0.026 | 0.026 | 1 | 241.28 | 0.4361 | 0.509634 |
| Exp_day × species | 2.649 | 1.325 | 2 | 410.47 | 22.4054 | **<0.0001** |
| Experiment × species | 0.173 | 0.087 | 2 | 241.28 | 1.4666 | 0.232763 |
| Treatment × species | 0.026 | 0.013 | 2 | 241.28 | 0.2226 | 0.800603 |
| Exp_day × Experiment × treatment | 0.127 | 0.127 | 1 | 410.47 | 2.14 | 0.144268 |
| Exp_day × Experiment × species | 1.416 | 0.708 | 2 | 410.47 | 11.9737 | **<0.0001** |
| Exp_day × treatment × species | 0.041 | 0.020 | 2 | 410.47 | 0.3453 | 0.708196 |
| Experiment × treatment × species | 0.162 | 0.081 | 2 | 241.28 | 1.3734 | 0.255216 |
| Exp_day × Experiment × treatment × species | 0.737 | 0.369 | 2 | 410.47 | 6.2346 | **0.002151** |
|  |  |  |  |  |  |  |
| Posthoc test on Exp_day × Experiment × treatment × species: | | | |  |  |  |
| treatment = Mono, species = *Amphidinium*: | | | | | | |
| Experiment: | Slope estimate | SE | Lower CL | Upper CL |  |  |
| Week 9 | -0.0730 | 0.01035 | -0.0933 | -0.0526 |  |  |
| Week 17 | -0.0407 | 0.01025 | -0.0609 | -0.0205 |  |  |
|  |  |  |  |  |  |  |
| contrast | estimate | SE | df | t ratio | p value |  |
| Week 9 – Week 17 | -0.0323 | 0.01457 | 411 | -2.215 | **0.0273** |  |
|  |  |  |  |  |  |  |
| treatment = Poly, species = *Amphidinium*: | | | | | | |
|  | Slope estimate | SE | Lower CL | Upper CL |  |  |
| Week 9 | -0.0913 | 0.00567 | -0.1025 | -0.0802 |  |  |
| Week 17 | -0.0653 | 0.00562 | -0.0763 | -0.0542 |  |  |
|  |  |  |  |  |  |  |
| contrast | estimate | SE | df | t ratio | p value |  |
| Week 9 – Week 17 | -0.0260 | 0.00798 | 411 | -3.263 | **0.0012** |  |
|  |  |  |  |  |  |  |
| treatment = Mono, species = *Dunaliella*: | | | | | | |
|  | Slope estimate | SE | Lower CL | Upper CL |  |  |
| Week 9 | -0.1193 | 0.01035 | -0.1397 | -0.0990 |  |  |
| Week 17 | -0.0522 | 0.01025 | -0.0723 | -0.0320 |  |  |
|  |  |  |  |  |  |  |
| contrast | estimate | SE | df | t ratio | p value |  |
| Week 9 – Week 17 | -0.0672 | 0.01457 | 411 | -4.610 | **<0.0001** |  |
|  |  |  |  |  |  |  |
| treatment = Poly, species = *Dunaliella*: | | | | | | |
|  | Slope estimate | SE | Lower CL | Upper CL |  |  |
| Week 9 | -0.1432 | 0.00567 | -0.1544 | -0.1321 |  |  |
| Week 17 | -0.0896 | 0.00562 | -0.1006 | -0.0786 |  |  |
|  |  |  |  |  |  |  |
| contrast | estimate | SE | df | t ratio | p value |  |
| Week 9 – Week 17 | -0.0536 | 0.00798 | 411 | -6.718 | **<0.0001** |  |
|  |  |  |  |  |  |  |
| treatment = Mono, species = *Tisochrysis*: | | | | | | |
|  | Slope estimate | SE | Lower CL | Upper CL |  |  |
| Week 9 | -0.0379 | 0.01035 | -0.0583 | -0.0176 |  |  |
| Week 17 | -0.0661 | 0.01025 | -0.0863 | -0.0459 |  |  |
|  |  |  |  |  |  |  |
| contrast | estimate | SE | df | t ratio | p value |  |
| Week 9 – Week 17 | 0.0282 | 0.01457 | 411 | 1.934 | *0.0538* |  |
|  |  |  |  |  |  |  |
| treatment = Poly, species = *Tisochrysis*: | | | | | | |
|  | Slope estimate | SE | Lower CL | Upper CL |  |  |
| Week 9 | -0.0978 | 0.00567 | -0.1089 | -0.0866 |  |  |
| Week 17 | -0.0640 | 0.00592 | -0.0756 | -0.0523 |  |  |
|  |  |  |  |  |  |  |
| contrast | estimate | SE | df | t ratio | p value |  |
| Week 9 – Week 17 | -0.0338 | 0.00820 | 411 | -4.123 | **<0.0001** |  |
|  |  |  |  |  |  |  |
